# Co‐Design Insights for Mechanically Active Flexible Printed Circuit Boards Through the CurveQuad Origami Robot

**DOI:** 10.1002/advs.78428

**Published:** 2026-10-29

**Authors:** Xuelin Wu, Daniel Feshbach, Satviki Vasireddy, Louis Beardell, Bao To, Yuliy Baryshnikov, Cynthia Sung

**Affiliations:** ^1^ General Robotics, Automation, Sensing & Perception (GRASP) Lab University of Pennsylvania Philadelphia USA; ^2^ Princeton Day School Princeton USA; ^3^ Episcopal Academy Newtown Square USA; ^4^ The Peddie School Hightstown USA; ^5^ Department of Mathematics and Electrical and Computer Engineering University of Illinois Urbana‐Champaign Urbana USA

**Keywords:** body‐circuit co‐design, embedded sensing, flexible printed circuit board, mechanically active fpcb, origami robotics, self‐folding, small‐scale robot

## Abstract

Folding flexible printed circuitboards (FPCBs) into origami patterns offers a route to fabricating robots with actuation, sensing, and control fully integrated into the robot body. However, integrating electronics into origami robots introduces a coupled electrical and mechanical design problem since the circuit layout affects the local stiffness and mass distribution and therefore the deformation mechanics. We explore this problem in the context of the FPCB CurveQuad, a centimeter‐scale curved‐crease origami quadruped. The robot body is fabricated as a folded double‐sided FPCB that contains the crease pattern, copper routing, sensors, local stiffening features, and electrical interfaces. A single servomotor drives tendons on the underside of the body, enabling the sheet to self‐fold from a planar state and twist for crawling and steering. The resulting robot is 60 mm × 65 mm × 50 mm in the folded state and weighs 10.9 g. It crawls at 16.8 mm $s^{-1}$ (0.259 body lengths $s^{-1}$), steers by shifting the actuator oscillation range, and is capable of feedback control. We present our design process for this robot, illustrating strategies for addressing electromechanical co‐design with complex bending requirements, enabling folded FPCBs to function as robotic mechanisms rather than simply as passive electronics substrates.

## Graphical abstract

This paper presents the process of integrating electronics directly into the body sheet of CurveQuad, a small origami robot that can self‐fold, crawl, and steer with a single actuator. The authors design a flexible printed circuit board for the body sheet: the key challenge is to navigate how embedding electronics affects the mechanical properties and thus the robot's functionalities.

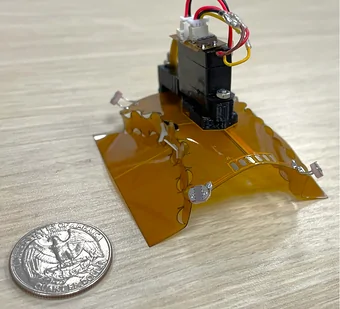

## 1 Introduction

Origami is an emerging platform for robot design because of its ability to leverage 2D manufacturing to construct 3D forms [1, 2, 3]. Robots constructed in this way have the benefit of high strength‐to‐weight ratios [4], can take advantage of patterns with parameterized stiffness [5, 6, 7], and leverage rapid planar fabrication techniques [8] potentially augmented with embedded electronics [9, 10] and self‐assembly via self‐folding [11, 12, 13, 14]. At the same time, origami‐inspired robots introduce significant design challenges since designing a functional robot now involves not only strategic placement in the final 3D form but also in the initial 2D design layout. This is particularly challenging at small scales, where volume and mass constraints call for efficient and multifunctional use of minimal components [15].

In this paper, we explore this multi‐functional integration in the context of the FPCB CurveQuad robot (Figure 1), a centimeter‐scale quadrupedal robot constructed from a flexible printed circuit board (FPCB). The robot is based on a previous design [16] where the curved crease pattern, combined with tendon‐driven actuation, enabled self‐folding, self‐unfolding, and steerable crawling, all driven by a single motor. The simple fold pattern and minimalist actuation amplify the mass manufacturability advantages of origami‐based design, while the functionality relies on body compliance from bending in shifting directions: this combination makes the design ideally suited for exploring both opportunities and challenges of directly embedding electronics and active components into the robot body. In this paper, we extend the design by constructing the body itself from an FPCB, exposing along the way the complex interactions between the FPCB design and other mechanical design features, particularly those involving body compliance. Our results highlight key interdependencies for mechanically active FPCBs and suggest strategies for navigating them, demonstrating how electronics can be embedded to achieve planar manufacturing of small‐scale compliant robots.

**FIGURE 1:**
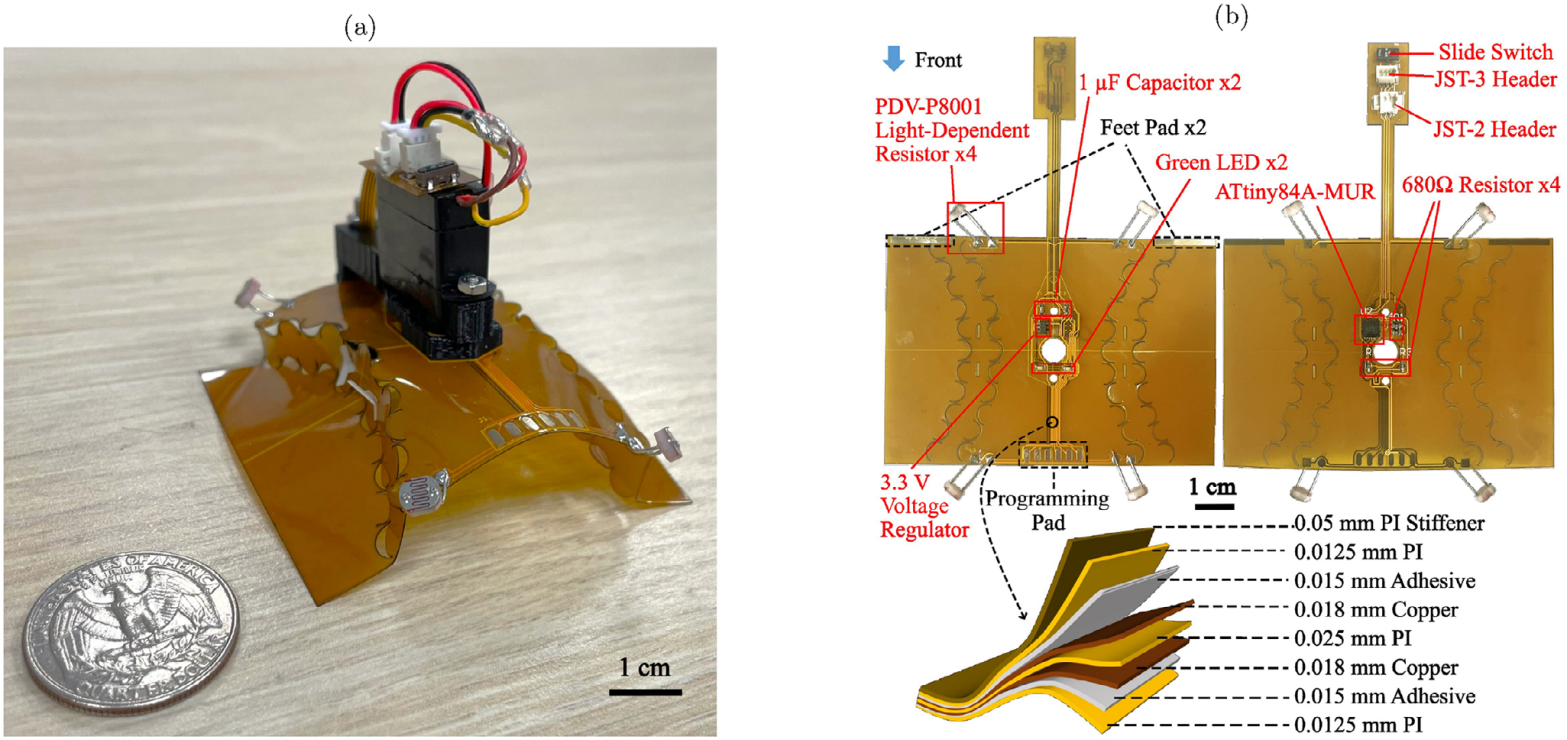
(a) CurveQuad in its folded neutral state. A U.S. quarter coin is included for scale comparison. (b) Top view (left) and bottom view (right) of the FPCB body sheet. The bottom cartoon shows a breakdown of the FPCB layers. Dashed lines indicate the copper pads used for the robot's feet, and solid lines show the electronic parts. The front of CurveQuad is the bottom of this figure.

### 1.1 Related Work

Embedding electronics into CurveQuad illuminates challenges involved in combining key benefits from several previous lines of work: design of origami‐inspired robots and curved‐crease mechanisms, walking and crawling robots at small scale with minimalist actuation, and mechanically active FPCBs.

#### 1.1.1 Origami‐Inspired Design

Origami involves folding and sculpting techniques to generate a 3D shape from flat sheets. Using existing planar fabrication and lamination techniques, origami‐inspired designs can directly embed electronics, actuators, and computing and allow 2D sheets to be rapidly assembled into 3D structures manufacturable in a matter of hours [7, 8, 11]. Self‐folding deployment of origami mechanisms has been demonstrated with a variety of techniques including heat‐based layer shrinking [11, 12], smart actuators embedded along creases [14, 17], chemical reactions [18], magnetic panels in external magnetic fields [13], and tendon‐driven systems [19].

Origami‐inspired robots have achieved tasks including jumping [20, 21, 22], walking [8, 11, 12, 23], swimming [6, 24], and manipulation [7, 25]. In the legged locomotion domain, origami has enabled rapid part fabrication [10, 26] and full‐body integration [8, 11, 12, 27, 28, 29], with some designs utilizing advanced methods including in‐plane embedding of electronics [10, 11, 29, 30], passive joint compliance [29, 31], or fully actuated linkages made of origami [26, 28].

Creases along curves, as opposed to the more typical straight creases, offer unique mechanical properties since they inherently bend the surrounding sheet, inducing complex energy storage such that just a few curved creases enable a wide range of deformation states [32, 33, 34]. As a result, the controlled release of the sheet allows a small number of actuators to generate coordinated and reversible deformations [19]. Prior research has examined the ways in which curved creases can distribute bending stiffness [35], adjust pattern bistability [36], create collapsible and deployable structures [19, 37, 38], and adjust the rigidity of devices such as a gripper and a swimming robot [6]. CurveQuad explores how tendons all driven by a single actuator can shift the bending direction to transition between a variety of folded states for steerable locomotion [16].

#### 1.1.2 Small‐Scale and Minimally‐Actuated Walking and Crawling Robots

Walking and crawling robots at the centimeter scale are promising tools for exploring or sensing in remote environments. Small‐scale robots can access confined spaces [23, 39, 40, 41, 42], operate with efficient energy consumption [43, 44, 45], and be manufactured with low material [46, 47, 48] and fabrication costs [49, 50]. These characteristics increase potential for deploying large numbers of robots where individual robots may need to be expendable, for example in search and rescue operations [23, 51] and environmental monitoring [52, 53].

To keep designs lightweight, low‐cost, and simple for mass manufacturability, it is desirable to get as much functionality as possible from as few actuators as possible. While implementing versatile gaits typically requires scaling the number of actuators with the degrees of freedom [26, 28, 31, 54], a few designs have achieved steerable walking or crawling with just a single actuator [16, 55, 56, 57, 58]. One strategy to achieve this is to use slip‐stick contact (e.g., from hooked feet on carpet [56] or fabric [58]) with a mechanism pulling one side's feet forward without the other moving backward; another strategy is to partition the actuator's timing [55] or range of motion [57] into distinct ground contact modes. CurveQuad combines aspects similar to each of these strategies: it achieves steerable crawling on flat surfaces through slip‐stick foot contact arising from its sheet bending and center of mass oscillation, while it additionally accomplishes self‐deployment using a different range of angles in the same actuator [16]. Each of these functionalities relies on shifting directions of sheet bending throughout actuation, which presents a major challenge in electronics integration.

#### 1.1.3 Flexible Printed Circuit Boards

To simplify fabrication and enable mass manufacturing, it is helpful for robot designs to be flat‐constructable with electronics embedded in plane. For compliant robots, however, it can be challenging to embed the electronics into a compact structure while maintaining both flexibility and reliability. To address this challenge, previous work has explored the use of integrated flexible structures as the main body, allowing for incorporation of components [13, 44, 59, 60, 61] while achieving a low mass and reduced thickness [39, 62, 63, 64]. Although flexible structures can be more complex to fabricate than rigid structures [65], flexible printed circuit boards (FPCBs) provide a mature technology solution with well‐established applications and capabilities for mass production [66]. FPCBs enable designs that are lightweight and adaptable to different shapes, making them useful for wearable devices [67, 68], space‐constrained sensors [63], and electrostatic actuators [69]. For crawling robots, using FPCBs as the body structure offers several advantages: they provide elasticity and mechanical robustness to withstand impacts [59], conform to uneven surfaces and overcome obstacles [54], and enable integration of custom and off‐the‐shelf components [39, 61, 62, 66]. Additionally, FPCBs allow electronic components to function under large deformations of the circuit [60], allowing them be folded and bent along crease patterns and therefore support integrated, low‐mass, and mass‐manufacturable origami‐inspired designs for actuators [70], actuated joints [71], stretchable electronics [72], and robots [14, 44, 73]. At the same time, when electronic circuits are integrated into deforming sheets, the conductive and substrate layers also become part of the mechanical structure. Previous work has demonstrated circuit boards that can maintain electrical function through repeated folding and three‐dimensional shaping [74]. These foldable integrated circuits use multilayer structures to control strain during large deformation [75], but the mechanical response and failure of the metal–polymer structure depend on the properties and thickness of the conductive layer [76]. These results suggest that when an FPCB itself bends and folds, its conductive layers and circuit structure should also be considered as part of the mechanical design.

### 1.2 Contributions and Implications

We showcase the potential for FPCBs to make active mechanisms with complex bending and built‐in electronics by extending the CurveQuad [16] design to construct the robot's body out of an FPCB. This is a challenging and illuminating testbed for a mechanically active FPCB because achieving all of its functionalities on a single actuator involves using a curved‐crease pattern where the body sheet bends and twists in multiple directions to store and release energy, subjecting the required functional behavior to highly coupled constraints between mechanical properties and electronics arrangement. We address these interdependencies in order to integrate electronic and structural components into a single FPCB CurveQuad design, allowing direct mounting for simpler manufacturing. The resulting FPCB CurveQuad's functions include self‐folding and self‐unfolding, crawling and steering on flat surfaces of different materials, sensor‐based feedback control, and multi‐robot behaviors.

Our work builds on previous research on FPCB‐based robots [11, 14, 73, 77] in which FPCBs form either the body or a supporting layer of the robot. These examples leverage the flexibility of the FPCB to create localized hinges or folds where the robot's body needs to flex, and a large focus lies in how to design the fold geometries to ensure that they fold reliably to the necessary angles without breaking the actuators or electronic traces [14]. The design breakdown in [77] emphasizes how this approach to robot design introduces significant co‐design challenges in mechanism design, material and actuator choice, and their effects on robot behavior. However, all of these existing projects focus on rigidly foldable patterns, that is, patterns that require deformation only at the folds and not along the faces of the robot, simplifying the design and modeling problem since rigid electronics can be placed on rigid faces without affecting the robot design. Our work builds beyond these examples to consider future electronics integration into thin‐sheet mechanisms relying on bending for functionality, such as robots formed from non‐rigid‐foldable origami patterns. At a basic level, our results demonstrate that mechanical requirements for specific and complicated sheet bending need not prevent the direct integration of electronics into mechanisms. More specifically, our design process and discussion identify key couplings where functionality is critically dependent on interactions between multiple design factors (including crease fabrication and layout, tendon arrangement, electronics placement, center of mass, and flexibility), and suggests strategies for navigating these interdependencies.

### 1.3 Outline

The remainder of this paper is organized as follows. Section 2 gives a high‐level overview of CurveQuad's design and functionality. Section 3 presents our iterative design process for the FPCB body, discussing the key relationships between design choices, design properties, and functionalities. Section 4 explores the effect of different circuit layouts on the robot's bending using the MERLIN2 [78] origami simulator. Section 5 shows our results: the final FPCB design, and experimental validation of its gaits and embedded sensing. Section 6 discusses principles learned from our design process that are transferrable to the design of other mechanically active FPCBs. Section 7 concludes with discussion of implications and future directions for CurveQuad and electromechanical co‐design.

## 2 CurveQuad Design Overview

CurveQuad (Figure 1) is a centimeter‐scale origami robot constructed from a pattern of four curved creases, which cause the sheet to self‐fold into an arched body that contacts the ground at the four corners. The fold pattern was previously described in [16], and we use the same pattern here in our FPCB design. The curved body design enables multiple deformations, including self‐folding from and return toward the flat state, as well as twisting of the body to create different contact modes. In the following discussion, we use *bending* to refer to deformation within the panels, *(self‐)folding* to refer to (self‐actuated) changes in the effective crease angles and resulting changes in the overall body shape (including bending^1^), and *deployment* to refer to the functional‐level transition (achieved by self‐folding) from the flat state to the operational state. To generate these deformations, a servomotor pulls on tendons underneath the body. When the tendons are pulled symmetrically, CurveQuad deploys into its standing configuration or returns toward the flat state. When the tendons are pulled diagonally, the robot twists. CurveQuad is designed such that the center of mass is behind the geometric center, inducing differential friction between the two back feet when the twisting causes the robot to lean sideways. Oscillation between left and right twisting creates slip‐stick crawling, where adjusting the center of oscillation controls the steering.

### 2.1 Body and Actuation System

The crease pattern, link, and tendon connections used for CurveQuad's deployment and locomotion are illustrated in Figure 2. The crease pattern consists of four symmetric parabolic creases on a rectangular sheet, with two on each side and all parabolas opening outward. The inner creases are mountain folds while the outer creases are valley folds, so actuating the creases causes the sheet to stand up and the robot to deploy. To produce this motion, we use a tendon‐driven mechanism whereby a servomotor rotates a straight, rigid link that pulls on tendons connected to the underside of the robot's body sheet. The link rotates about the robot's z axis (which is normal through the body sheet's center). Let θ denote the angle of link rotation, measured clockwise from the −y direction in the top view. Four tendons connect the two *link endpoints* $e^{+},e^{-}$ each to two of four *attachment points* $s^{L+},s^{L-},s^{R+},s^{R-}$ as diagrammed in Figure 2 and photographed in Figure 3b: L and R are left and right, + and − are front and back respectively in the unfolded state $\theta =180^{\circ }$. Mathematical details of the crease pattern and link‐tendon geometry are included in Section S1.1 (including Figure S1 illustrating geometric parameters) and Section S1.2.

**FIGURE 2:**
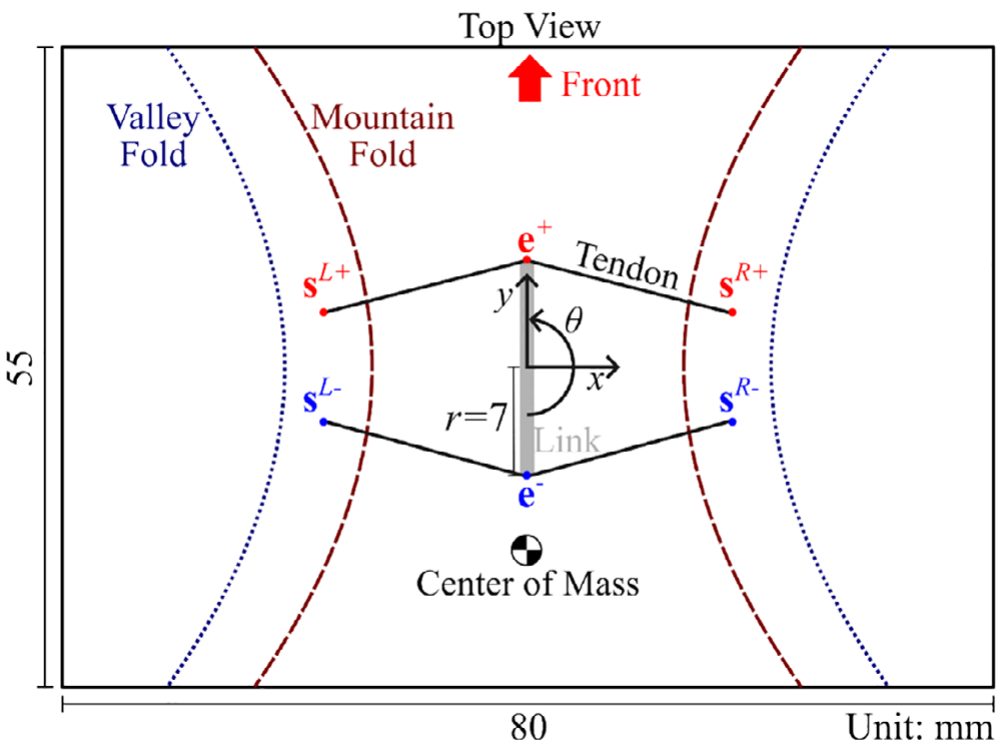
Top view diagram of the crease pattern and link‐tendon system at the unfolded state $\theta =180^{\circ }$. The inner creases are mountain folds and the outer creases are valley folds. The link is parallel to and below the plane of the sheet. The sheet plane lies below the center of mass (CoM). The red arrow on the top shows the front direction of CurveQuad.

**FIGURE 3:**
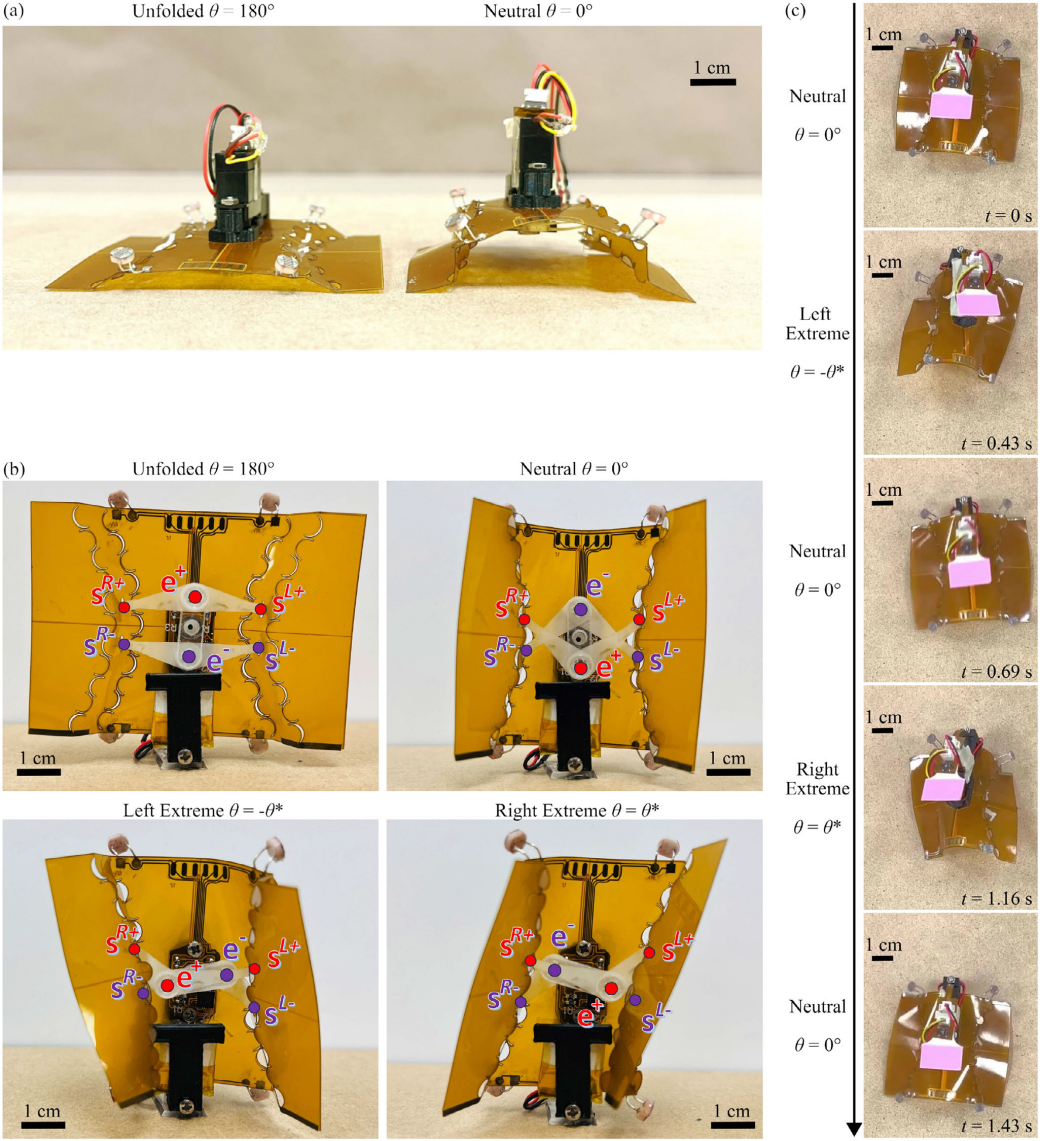
The robot's key states and how they sequence into a forward gait. (a) Front view of unfolded and neutral states. (b) Four key states of robot shown from the underside view. Tendon endpoints and link endpoints are annotated. Note that $s^{L+}$ and $s^{L-}$ appear on the right, and vice versa, due to the underside view. (c) Snapshots of the robot crawling forward, starting from the neutral state. The 1 cm scale bar is placed at the same location in every image for comparison.

### 2.2 Deployment and Crawling

The robot can achieve deployment and crawling motions by deforming its body along the curved creases using the tendon mechanism. Figure 3 shows several body states that are used for these motions. Movie S1 shows CurveQuad self‐folding and walking from four views. The states can be defined by the rotation angle θ of the servomotor‐driven link. CurveQuad has four key states: *unfolded* ($\theta =180^{\circ }$), *(folded) neutral* ($\theta =0^{\circ }$) with left‐right symmetry, *(folded) left* ($\theta \in [-\theta^{*},0^{\circ })$), and *(folded) right* ($\theta \in (0^{\circ },\theta^{*}]$). There is a natural *extreme angle* $\theta^{*}\approx 75^{\circ }$ maximizing contraction for one diagonal pair of tendons by aligning the link between the corresponding sheet attachment points: we call the $\theta =\pm \theta^{*}$ states the *(folded) left extreme* and *(folded) right extreme* states. By alternating between these states in preset patterns, the robot is able to deploy, shuffle forward, and turn on flat ground.

Self‐folding is achieved because the transition from $\theta =180^{\circ }$ to $\theta =0^{\circ }$ swaps the position of the link endpoints and brings them farther away from the original positions of their corresponding sheet attachment points, so the tendons symmetrically pull those points inward (Figure 3a). Whenever $\theta \notin \{0^{\circ },180^{\circ }\}$, one diagonal pair of tendons is pulled more than the other pair, inducing bending and twisting of the robot and thereby moving the feet. With $\left|\theta \right|\leq 90^{\circ }$, both diagonal pairs are still sufficiently pulled to keep the creases folded and the robot standing upright.

A *gait* is an oscillation between a pair $\left(\theta_{L},\theta_{R}\right)$ of angles, with $\theta_{L}\in \left[-\theta^{*},0^{\circ }\right]$ and $\theta_{R}\in \left[0^{\circ },\theta^{*}\right]$. The middle angle of a gait steers the gait left or right, while the gait's angle range scales the magnitude of displacement. So the *left extreme gait* (moving forward while turning leftward as much as possible) is $\left(-\theta^{*},0\right)$, the *right extreme gait* is $\left(0,\theta^{*}\right)$, and the fastest *forward gait*, depicted in Figure 3c, is $\left(\theta_{L},\theta_{R}\right)=\left(-\theta^{*},\theta^{*}\right)$: this alternates left‐extreme and right‐extreme steps where both move the robot forward and their rotations cancel out. Detailed gait‐state definitions are provided in Section S1.3.

## 3 FPCB‐Integrated CurveQuad as a Design Validation Platform

While the high‐level design of CurveQuad is relatively simple, fully embedding functionality into the origami pattern involves complex interactions between pattern, material, actuator, and controls decisions. The robot must satisfy a combination of functionality‐ and fabrication‐related constraints. In functionality, the original curved‐crease geometry and tendon‐driven actuation system already define the robot's core body functions: the sheet must self‐fold from a planar state, unfold back toward the flat state, twist between left‐ and right‐biased configurations, and support crawling and steering through ground contact. In fabricability, the design must be able to be fabricated as a flat sheet, components soldered or otherwise attached orthogonally to the body, and resulting sheet folded into the desired 3D shape. These two requirements interact in that the fabrication choices affect the folded 3D shape and the achievable deformations. In particular, the robot body becomes a coupled electromechanical structure with the FPCB participating directly in the same folding, twisting, support, and foot‐contact functions that generate robot motion.

In previous work [16], functionality and fabricability were decoupled physically so that the main deforming body of the robot was constructed from PET film and the circuitry and electronics lay separately on top. In this section, we outline the design constraints and design strategies associated with fully integrating the two so that the body sheet is redesigned as a flexible printed circuit board that also carries the electronics, sensors, power routing, and actuator interfaces. As a result, circuit layout decisions, including stackup, copper routing, component placement, connector location, and local deformation, also become mechanical design decisions. We first organize the coupled body–circuit requirements into a design dependency map, linking controllable design choices to intermediate mechanical properties and robot‐level functions. We then describe the prototype iterations that identified the main conflicts between FPCB integration and the deformation modes required for deployment, crawling, and steering.

### 3.1 Design Requirements and Dependency Map

Figure 4 summarizes the main dependencies that must be considered when the CurveQuad body is redesigned as an FPCB. The map is organized in three levels: design choices, intermediate body properties, and robot‐level functions. The design choices include the crease geometry, tendon arrangement, material and stackup, component arrangement, and copper arrangement. These choices are directly specified during the body–circuit design process, but they do not determine robot behaviors independently. Rather, they modify intermediate properties of the body, including the center of mass, bending energy storage and release, stiffness distribution, and ground contact. These body properties then determine whether the robot can preserve its main functions: steering, slip‐stick foot motion and net forward displacement, stable upright stance, and self‐folding/deployability.

**FIGURE 4:**
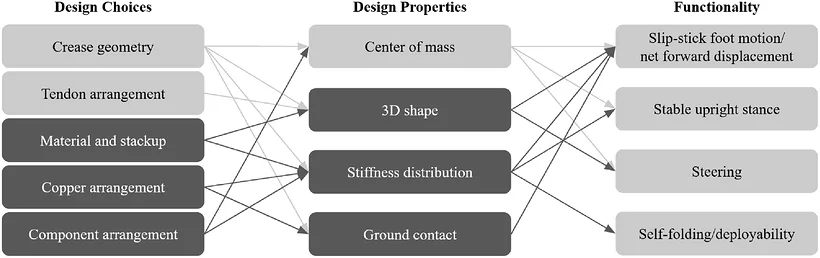
Design dependency map for the FPCB‐integrated CurveQuad. The dark boxes indicate the choices and properties that are most strongly affected by replacing the passive body sheet with an FPCB body: the goal of the design process for FPCB integration is to manage changes among these dependencies to preserve the functionalities.

The crease geometry and tendon arrangement define the deformation space of the robot. The curved creases determine where bending and folding are allowed, and therefore affect both the stiffness distribution and the bending energy stored during folding and twisting. They also determine the folded body shape, which affects the angles and contact areas of the feet. The tendon arrangement determines how the servo input is transmitted into the body. Symmetric tendon pulling produces self‐folding and deployability, while diagonal contraction produces the left–right twisting used for slip‐stick foot motion and steering. Therefore, the crease geometry and tendon arrangement mainly influence the body through bending energy storage and release, stiffness distribution, and ground contact.

The FPCB‐specific choices enter the map through a different set of effects. The material and stackup determine the baseline flexibility of the body sheet and the local fatigue behavior near the crease cuts. The copper arrangement changes the local stiffness even when no electrical components are present, because trace width, trace direction, and whether copper appears on one or both sides of the sheet affect how easily the body twists. The component arrangement adds localized rigid regions and shifts the center of mass. These effects are especially important because the robot requires compliance in the high‐curvature regions, but stiffness in the central support region and in the feet. Thus, the same design feature can either support or suppress robot function depending on where it is placed.

For this reason, the dependency map should be read as a design guide rather than as a sequential pipeline. The design choices on the left do not map one‐to‐one to the functions on the right. Instead, each choice modifies several intermediate body properties, and each body property affects several robot functions. For example, stiffness distribution affects self‐folding/deployability because the body must be compliant enough to achieve the bending required for folding, stable upright stance because the body must support the servo and battery, and slip‐stick foot motion because the feet must remain in effective contact with the ground. Similarly, the center of mass affects both steering and net forward displacement by determining which feet dominate the ground interaction during body twisting.

The design objective is therefore to place stiffness, mass, and rigid interfaces where they support the desired body mechanics, while keeping high‐curvature and twisting regions clear. In the final design, this principle led to narrower and more symmetric copper routing, relocation of components toward mechanically quieter regions near the servomotor, local copper stiffeners at the rear feet, and an extension tail enabling some components to be placed on top of the servomotor. The following subsection describes how these dependencies were identified through prototype iterations.

### 3.2 Iterative FPCB Design and Prototype Evolution

The dependency map in Figure 4 was developed through a sequence of prototypes and controlled comparison tests, summarized in Figure 5. We organize these iterations into four stages. Each stage addressed a specific design question: whether the original PET body could be directly converted into an FPCB body, which FPCB features caused the first failure, how the FPCB layout could be reorganized to preserve the required deformation modes, and how rigid interfaces could be relocated without introducing new failure points.

**FIGURE 5:**
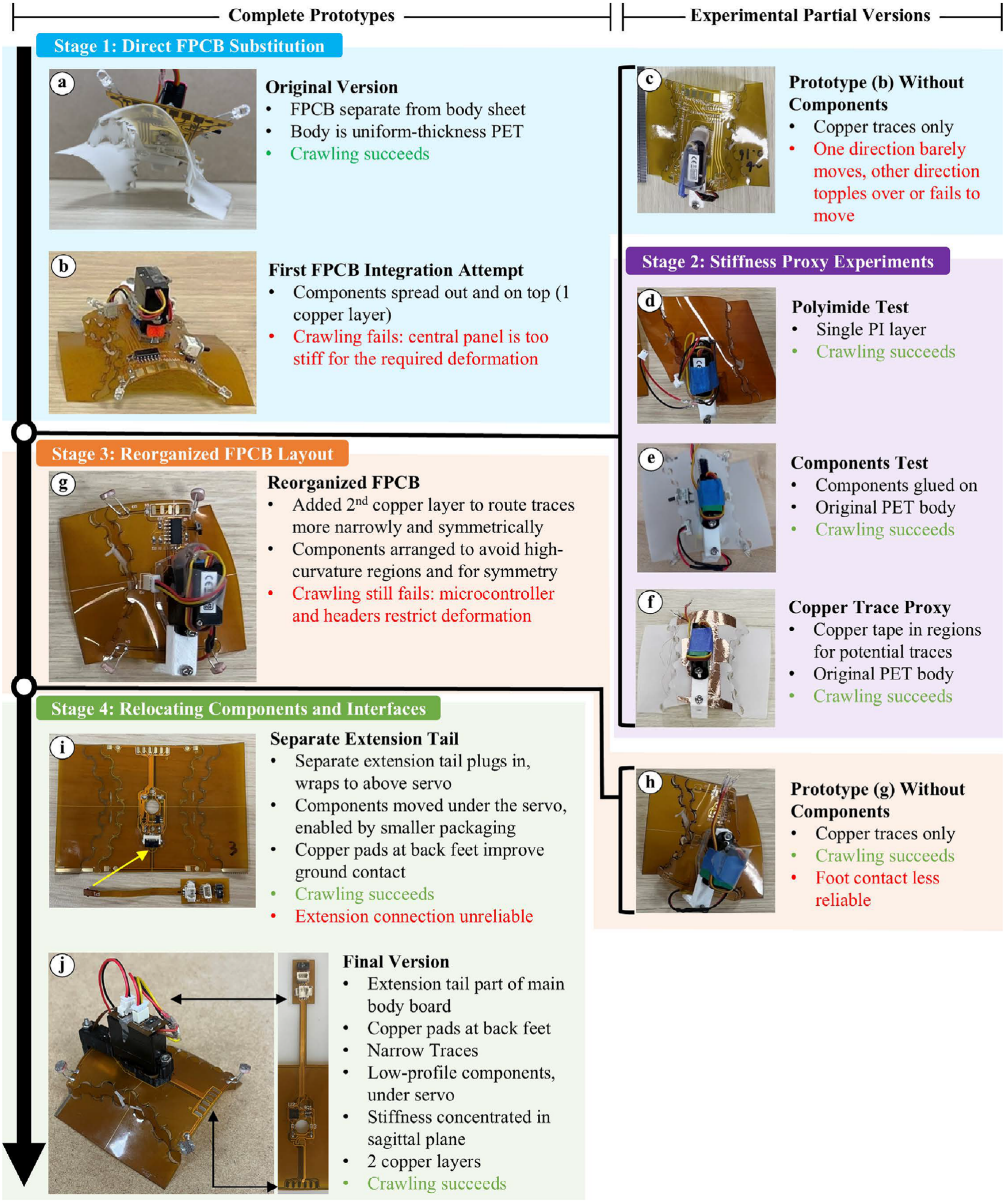
Design timeline showing high‐level stages (numbered chronologically) and specific iterations (alphabetized chronologically) with key changes summarized. All versions have the same crease pattern, size, and actuation system. Left column (a,b,g,i,j): major prototypes with all systems onboard. Right column (c,d,e,f,h): experimental versions without complete circuits, tethered to external power and control, for characterizing the stiffness impacts of particular subsystems.

#### 3.2.1 Stage 1: Direct FPCB Substitution

The first stage concerned whether the original CurveQuad architecture could be directly converted into an FPCB body. The previous PET‐based version (Figure 5a) established that the curved‐crease pattern and tendon‐driven mechanism could deploy, crawl, and steer. In that design, however, the electronics were mechanically separated from the main deforming body sheet. The first FPCB‐integrated attempt (Figure 5b) used a similar circuit layout, but made the body sheet itself an FPCB. Components were spread across the body in an attempt to distribute stiffness roughly evenly. This direct substitution failed to preserve the required self‐folding and twisting behavior. The FPCB was not mechanically equivalent to PET: the copper traces, FPCB stackup, and top‐mounted components made the central body too stiff and interrupted the deformation modes needed for crawling. Further experiments showed that the design without the rigid electronic components soldered on (Figure 5c) still failed to crawl, indicating that the copper traces themselves introduced sufficient stiffness variation to interfere with functionality. This stage showed that direct FPCB integration cannot be treated as a simple material substitution. Instead, the circuit layout must designed as part of the mechanical body.

#### 3.2.2 Stage 2: Stiffness Proxy Experiments

In the second stage, we designed proxy experiments to identify which factor caused the failure: the polyimide (PI) base layer sheet, the copper traces, or the rigid electronic components. A PI‐only body tested whether the base sheet material itself was the problem (Figure 5d). A PET body with glued component proxies tested whether rigid component packages could cause local bending constraints (Figure 5e). A PET body with copper‐tape traces tested whether conductive traces alone could disturb twisting (Figure 5f). These were intended as binary experiments to isolate individual factors. Each proxy version preserved basic actuation. While the proxies were low‐fidelity approximations of their real counterparts' effects on stiffness, these results suggested that the initial failure may not be from any specific one of these factors but from more spatially complicated interactions. Looking closer at how all the versions operated, we observed spatial distributions of bending curvature differed significantly between successful and unsuccessful versions. The successful bodies maintained continuous curvature through the active deformation regions, whereas the unsuccessful FPCB body developed locally rigid regions that disrupted folding and twisting.

These observations led to three design conclusions. First, the spatial distribution of stiffness matters, so stiffness should be distributed strategically rather than uniformly when designing the circuit. Second, the observed bending curvature indicates where flexibility is required and where additional stiffness can be tolerated. In particular, the servomotor and its mount already make the central region relatively rigid, and this rigidity does not prevent CurveQuad from functioning. This suggested that traces and components could be concentrated under or near the centerline where the servo is already placed. Third, many individual components are large enough to strongly affect local stiffness, so simply spreading them out is not sufficient to ensure uniform behavior. To preserve left–right symmetry in locomotion, the circuit design should also remain approximately left–right symmetric. This suggested the sagittal plane as a candidate region for concentrating traces and components.

The proxy tests also exposed local robustness issues. Compared with PET, the FPCB/PI body was more prone to tearing at sharp corners, narrow bridges, and edge features. These observations later motivated rounded tendon attachment holes, regularized arc‐cut spacing, and removal of weak partial arcs near the sheet edges, as discussed in Section 6.3.

#### 3.2.3 Stage 3: Reorganized FPCB Layout

In the third stage, we redesigned the FPCB layout to reduce mechanical interference from the circuit itself based on the proxy experiments. The goal was to test whether an FPCB body could preserve the required deformation modes if the traces and components were placed according to the mechanical behavior observed in Stage 2. Since the servomotor already created a relatively rigid region around it, we moved components nearby, enabling us to keep regions with higher curvature and twisting clear. We also added a second copper layer to the FPCB in order to enable more narrow and symmetric trace routing. However, when we tested the reorganized FPCB (Figure 5g) the crawling still failed as the body could not achieve adequate deformation. The deformation failure seemed to be from insufficient curvature around the largest rigid components. When we tested this prototype with tethered control without components soldered on (Figure 5h) it operated successfully, though with less stable and effective ground contact while pushing forward than the original version. The difference seemed to stem from the new version having more bending curvature at the back feet. This indicated that the local rigidity from components and local stiffness at the back feet were the main remaining problems. Additionally, comparing it to the failure of the initial board without components (Figure 5c) demonstrated that the two‐layer FPCB's narrower and more symmetric trace distribution successfully arranged stiffness, and more broadly that it was possible to make the FPCB stackup and copper routing mechanically compatible with the CurveQuad body.

This stage showed that the failure was not caused by the FPCB material or copper traces alone. The key issue was the interaction between copper routing, component placement, and local curvature. A working FPCB body therefore requires both mechanically compatible trace routing and component placement that avoids active deformation regions. For the subsequent design, this motivated using smaller low‐profile SMD packages and keeping high‐curvature regions clear, concentrating added stiffness near the sagittal plane.

#### 3.2.4 Stage 4: Relocating Components and Interfaces

The final stage of design addressed the remaining rigid elements that were difficult to place on the active body, including large headers, the power switch, the battery connector, and the servo connector. These interfaces are mechanically rigid and occupy substantial circuit area, so placing them on the twisting body would reintroduce local stiffness constraints. To reduce their interference with body deformation, we moved some components above the servomotor and connected them with an extension tail bending upward from the main board. Specifically, the components we placed above the servo were the switch and the servo and battery headers: we selected selected those components because they are touched during operation and maintenance, so we want them to be easily accessible. This provided an additional benefit to robustness, because having them up against a rigid backing protected their soldered connections from contact force. Most of the remaining components, most importantly the microcontroller, were moved underneath the servomotor, where the body was already fully constrained by the servo mount (fitting them in this limited surface area was made possible by switching to smaller SMD packages with no leads or lower profiles). In this stage, we also increased the stability of ground contact (based on the excessive curvature observed at the back feet in version (h)) by adding copper pads along the back feet: these are electrically inactive and serve only as a local stiffener.

Initially we implemented the extension tail as a separate piece plugging into a header behind the servomotor (Figure 5i). This restored successful crawling, but introduced a new weak mechanical and electrical connection between the main body and the tail. The final version therefore made the extension tail part of the same manufactured FPCB (Figure 5(j)).

Overall, compared with the first Stage 1 design, the final design uses a two‐layer FPCB body, narrower traces and smaller low‐profile components, components and traces placed near or along the axis of symmetry, an integrated extension tail, and local stiffness features near the feet. The curved‐crease pattern and tendon‐driven mechanism from the original robot are preserved, but the electrical system is relocated into regions where its stiffness, mass, and interfaces are compatible with, or in some cases directly support, the deformation modes required for deployment, crawling, and steering.

## 4 Effect of Circuit Layout on Pattern Deformation

In order to quantitatively explore the effect of the circuit layout on the body of the robot, we conduct a simulation analysis in the MERLIN2 [78] origami simulator. The purpose of this analysis is to isolate the mechanical effect of the spatial stiffness distribution introduced by copper routing. We therefore compare body deformation under different idealized stiffness distributions while holding the crease pattern, actuation condition, and baseline sheet properties constant. The simulation is intended to quantify the relationship between circuit layout and body deformation rather than directly predict robot‐level displacement, which additionally depends on the robot's complex slip‐stick interaction with the ground.

The CurveQuad body is represented using a bar‐and‐hinge model based on the curved‐crease geometry described in Figure 2. In order to capture differences in behavior resulting from different copper distributions, a 22×22 grid is overlaid over the pattern to create a higher resolution mesh.

To simulate different copper distributions, baseline material properties equivalent to the PI stackup without copper (experimentally measured to have a Young's modulus of 1.55 GPa, reported in Table S2 in Section S2.5) are first assigned to all of the elements. Then, hinges in regions containing copper are manually modified to increase their bending stiffness by 77.8 times, as determined by matching the flexural rigidity EI of the 0.130 mm PI layer and two 0.018 mm copper layers to the original homogeneous 0.130 mm layer. The stiffness calculation is provided in Section S5.2, 2.

We compare six stiffness distributions: no added stiffness, horizontal stripes, vertical stripes, an I‐shaped pattern, a +‐shaped pattern, and a grid pattern. Figure 6 shows the stiffness layouts, as well as the simulated results for the folded neutral and extreme configurations for each stiffness distribution. Details on how the simulation is set up for each of these configurations are in Section S5, with the tendon attachment to the mesh illustrated in Figure S5. Stiffness layouts are colored according to the assigned bending stiffness, and 3D configurations are colored according to mean curvature. Mean curvature is computed at each vertex using [79],^3^ then interpolated across faces.

**FIGURE 6:**
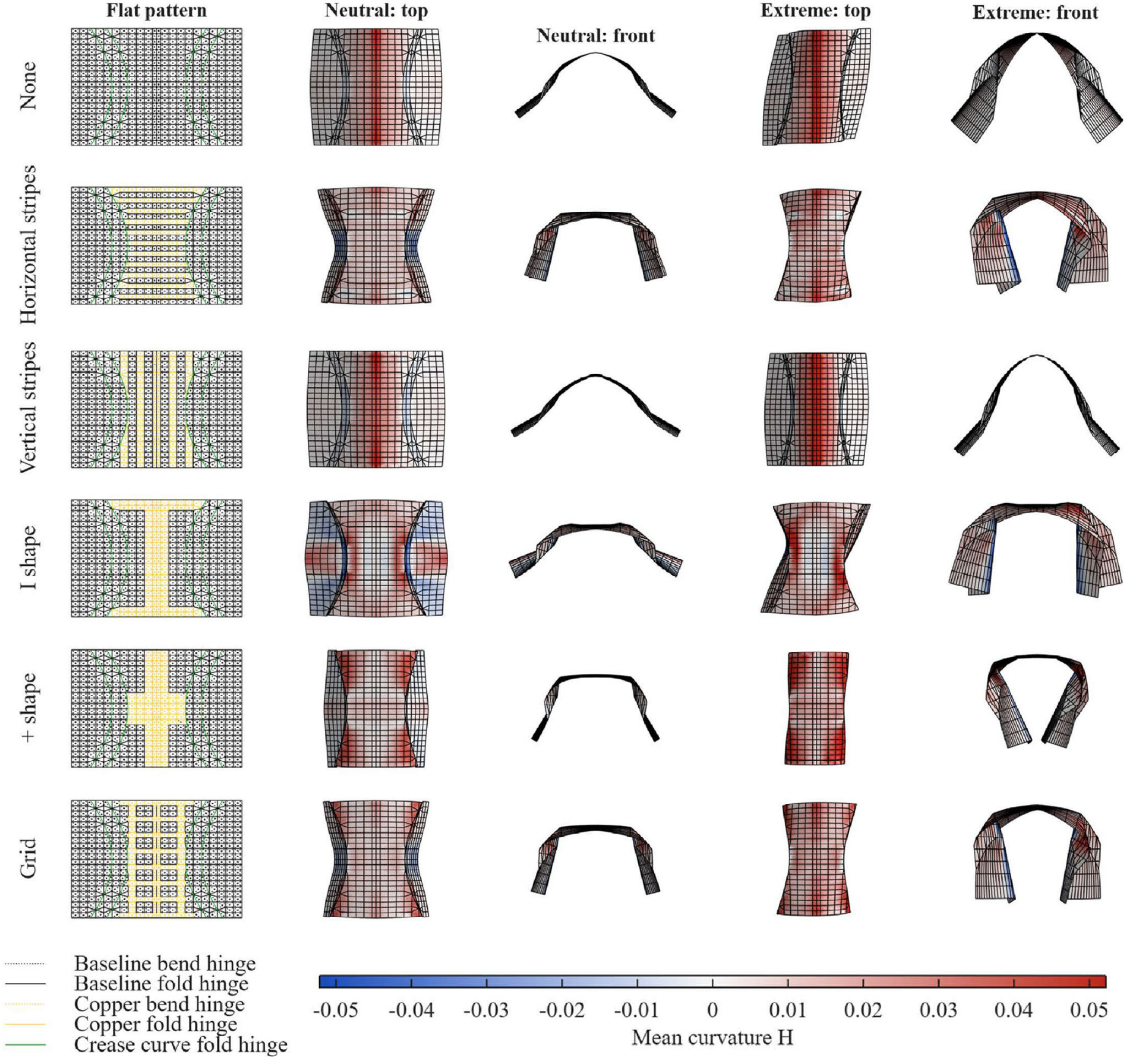
Effect of spatial stiffness distribution on CurveQuad deformation in the MERLIN2 simulations. Rows correspond to the six tested stiffness distributions: None, Horizontal stripes, Vertical stripes, I shape, + shape, and Grid. The first column shows the flat pattern and locations of the stiffened hinges. The remaining columns show top and front views of the simulated folded neutral and extreme configurations. Surface color indicates mean curvature, calculated separately over the five surface sections separated by the four curved creases.

The stiffened patterns are designed to have comparable coverage of the central panel while varying the spatial arrangement and orientation of the stiffened regions. Because the stiffness patterns are discretized onto the simulation mesh, the exact coverage varies slightly between cases; the values are listed in Table S4. Despite having similar amounts of added stiffness, the different spatial distributions produce distinct deformation profiles. In the neutral configuration, the PI‐only case produces a relatively smooth arch across the body. Adding horizontal stripes makes the central portion of the body noticeably flatter and shifts bending toward more localized regions, whereas the vertical‐stripe case retains a more continuously curved profile similar to the unstiffened case. This difference shows that the orientation of the stiffened regions, rather than only their total amount, determines where curvature can develop.

The I‐shaped distribution produces a different compromise. Its stiff central region remains relatively flat in the neutral configuration, while the surrounding regions continue to bend. In the extreme configuration, the body also retains a large asymmetric deformation, indicating that concentrating stiffness near the sagittal plane does not prevent the twisting motion required for the left and right gait states. In contrast, layouts that distribute stiffness more broadly across the active body, such as the horizontal‐stripe and grid patterns, constrain or redistribute bending over a larger portion of the sheet.

These differences connect directly to the CurveQuad design objectives identified in Section 3. The central region must provide structural support for the servomotor and other rigid components, while the surrounding body must remain compliant enough to fold and twist. Stiffness near the feet can additionally support stable ground contact. Therefore, the goal is not to minimize stiffness everywhere, but to concentrate it in mechanically less active or structurally useful regions while keeping the high‐curvature regions compliant. The I‐shaped stiffness distribution captures this design strategy by concentrating stiffness near the sagittal plane while leaving much of the lateral body available for deformation. The final CurveQuad circuit follows the same principle by concentrating traces and components near the sagittal plane and adding localized copper stiffening at the rear feet.

## 5 FPCB CurveQuad Design and Evaluation

Our FPCB design process results in a version of CurveQuad where the electronics are directly integrated into the body and all functionalities are preserved. In this section we describe the final FPCB design and robot fabrication (Section 5.1) and then present our experiments validating the functionality of gaits and embedded sensing (Section 5.2).

### 5.1 Design and Fabrication

The final body sheet is a multi‐layer polyimide (PI) circuit board with two copper layers and components mounted on both sides of the board. The layer structure, depicted in Figure 1b, was chosen to achieve a total thickness of 0.130 mm where there is no copper (close to the 0.127 mm thickness of the PET sheet in the original version). Where both copper layers have traces, the total thickness is 0.166 mm. The traces are 0.32 mm wide. Additional circuit and component details are provided in Section S2.1.

The circuit layout and components are depicted in Figure 1b. The circuit and servomotor are powered by a 3.7 V LiPo battery. The robot is controlled by an onboard microcontroller and receives sensing input from four photoresistors placed near the corners of the central panel. These sensors allow the same body sheet that generates locomotion to also perform feedback‐based behaviors.

Most components are placed underneath the servomotor or on top of it through a ribbon‐like extension tail that curls upward from the back of the FPCB to the top of the servo. The photoresistors are located at the corners of the central panel and face outward or upward (depending on the sensing needs of each task) to detect light gradients, while the programming pads are placed along the front center for clamp‐based programming. The traces to these locations are concentrated along the sagittal plane as much as possible to minimize interference with body twisting.

Additional electrically inactive copper pads along the back edge act as local stiffeners at the feet to increase the surface area of ground contact.

The FPCB stackup is manufactured by a commercial supplier (PCBWay), followed by crease and hole cutting and component mounting performed in our lab. The creases are manufactured as a series of alternating arc cuts, depicted in Figure 7a, leaving narrow bridges diagonally across the crease lines that bend and twist during folding. This allows repeated folding and unfolding with negligible plastic deformation along the crease. The cut pattern is slightly altered from [16], with the outermost partial arcs removed and the arc spacing made precisely regular. Additional fabrication details are provided in Section S2.2.

**FIGURE 7:**
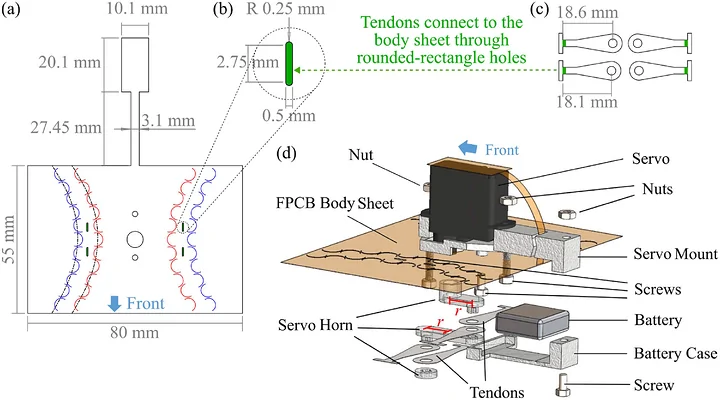
The body sheet's (a) laser‐cut pattern. The curved cuts reduce stress while constraining the pattern to fold along the crease (indicated by the dot‐dash line). The outer arcs (blue) are the cuts creating the valley folds, while the inner arcs are the cuts creating the mountain folds. The blue arrow shows the front direction of the robot. (b) Zoomed‐in view of the holes in the sheet through which the tendons attach. (c) Laser‐cut patterns for the tendon: the green shaded region indicates where they insert into the sheet holes. (d) Design assembly in exploded view. The blue arrow on the top of the servomotor shows the front direction of CurveQuad.

The actuation system for CurveQuad is unchanged from the original. Tendons (Figure 7c) are laser cut from 0.127 mm PET and attached to a servo horn printed in three pieces as shown in Figure 7d. The other ends of the tendons connect to the body sheet through holes (Figure 7b) whose corners have been rounded to minimize risk of tearing. The servomotor is attached to the sheet using a rigid mount, slightly modified from the structure in [16] to accommodate components mounted underneath it and the extension tail routed upward through it. The battery is held under the sheet in a case connected to the servo mount behind the body sheet. Overall, the components stack vertically, as shown in Figure 7d, to support pick‐and‐place mass manufacturability. Details of the mount, printed parts, and assembly process are provided in Sections S2.2 and S2.4, including a mass breakdown in Table S1.

After FPCB fabrication, the assembly process includes soldering the electronic components, attaching the servomotor and battery mount, inserting the tendons into the body slots, connecting the tendons to the servo horn, routing the rear FPCB extension, and connecting the battery, switch, and servomotor. The final robot is approximately 60×65×50 mm in the folded state and 80×65×40 mm in the unfolded state, including the attached electronics. It weighs 10.938 g in total. With a fully charged battery, the robot operates for approximately 55 min before its performance visibly degrades due to battery depletion. After recharging, normal performance is restored. We operated the same robot for two full battery‐discharge sessions, totaling over 5000 gait cycles, without observable mechanical failure. The assembly and programming procedures are shown in Movies S2 and S3, respectively.

### 5.2 Experimental Validation of CurveQuad Functionalities

The fabricated robot is capable of transitioning between the key states needed for CurveQuad's deployment and locomotion (Figure 3). To validate its functionalities, we conduct a series of experiments to characterize the performance of its gaits and demonstrate behaviors enabled by the embedded sensing. Except where otherwise noted, tests are conducted on a medium‐density fiberboard (MDF) surface. Videos of certain experiments are available, listed in Table S5.

#### 5.2.1 Effect of Tendon Attachment Location on Body Curvature

To examine how the amount of body bending affects robot functionality, we vary the tendon attachment parameter $s_{y}$ while holding the final FPCB layout and other body parameters constant. As defined in Section S1.2, $s_{y}$ is the common magnitude of the y‐coordinates of the four tendon attachment points on the body sheet. The same parameter was previously varied in the original PET CurveQuad [16], where an intermediate attachment position was also found to be necessary for successful deployment and locomotion. Here, we repeat this comparison using the final FPCB robot to determine whether the same dependence on body bending remains after electronics integration. We tested three attachment positions, $s_{y}=2$, 4.7, and 7 mm, spanning configurations with insufficient, intermediate, and excessive body bending.

As shown in Figure 8, the three attachment positions produce visibly different amounts of body bending. With $s_{y}=2$ mm, the body does not bend sufficiently to lift the battery case and actuation system from the ground, and the robot does not discernibly move forward during the gait. With $s_{y}=7$ mm, the body bends excessively, making the robot unstable and causing it to topple over when attempting to deploy or walk. The intermediate $s_{y}=4.7$ mm configuration produces sufficient bending to keep the robot upright with the body corners contacting the ground and supports stable deployment and locomotion. This value is therefore used for the functional robot throughout this work.

**FIGURE 8:**
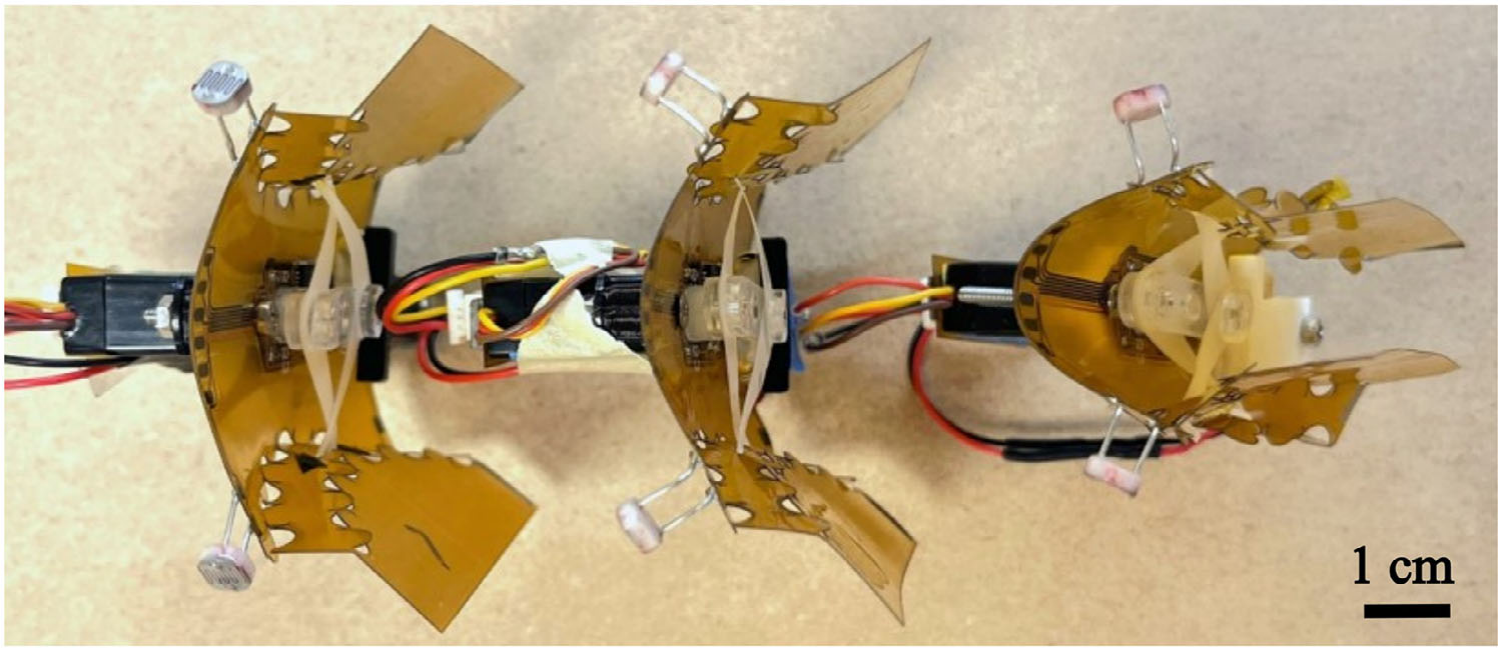
Effect of the tendon attachment parameter $s_{y}$ on body curvature in the final FPCB CurveQuad. The robots are shown in the neutral state ($\theta =0^{\circ }$) with $s_{y}=2$ mm (left), $s_{y}=4.7$ mm (center), and $s_{y}=7$ mm (right). The FPCB layout is held constant across the three versions.

Complementing the FPCB design iterations in Section 3.2, this experiment provides an additional example of design parameters affecting bending in ways that determine robot functionality. In the FPCB design history, the initial FPCB without rigid components (Figure 5c) failed to crawl, whereas the reorganized FPCB without components (Figure 5h) operated successfully. Because these versions differed in the FPCB layout while excluding the effects of soldered rigid components, their comparison showed that changes in the circuit layout can alter the body deformation sufficiently to determine whether the robot functions. In the tendon‐attachment experiment here, the FPCB layout is instead held constant, while body bending is varied through $s_{y}$. Together, these two sets of observations show that appropriate body bending is critical to CurveQuad functionality, whether it is altered by the FPCB layout or by the tendon geometry.

#### 5.2.2 Gait Characterization

We measure the gait performance across two main types of variation: gait parameters steering the trajectory and behavior consistency across fabricated robots. Additional experiments varying starting location (Figure S2 in Section S3.2) and surface material (Table S3 in Section S3.3) are provided in Supporting Information.

Gait experiments are conducted by tracking the center of mass with motion capture and extracting its 2D displacement and heading change at corresponding points in consecutive gait cycles. We run three trials per gait and measure motion from 10 cycles per trial. Additional details of the motion capture setup and data processing are in Supporting Information (Section S3.1).

To measure robot motion, we test the 8 gaits from pairwise combinations of $\theta_{L}\in \left\{-\theta^{*},-\theta^{*}/2,0^{\circ }\right\}$ and $\theta_{R}\in \left\{0^{\circ },\theta^{*}/2,\theta^{*}\right\}$ (excluding the empty gait (0∘,0∘)), where $\theta^{*}\approx 75^{\circ }$ is the extreme angle (Section 2.2). The resulting trajectories are shown in Figure 9a, the average displacement and rotation per cycle are summarized in Table 1, and a video grid showing representative trials for the tested gait parameters is provided in Movie S4. Consistent with the original version, the robot's displacement increases with the angular range $\theta_{R}-\theta_{L}$ of the gait, while steering depends on the directional bias between $\theta_{L}$ and $\theta_{R}$. These results validate that the FPCB‐integrated body preserves controllable locomotion after electronic and structural integration. The forwardmost gait $\left(-\theta^{*},\theta^{*}\right)$ averages 21.7 mm displacement (corresponding to a speed of approximately 16.8 mm/s or 0.259 body lengths per second). The leftmost gait $\left(-\theta^{*},0\right)$ averages rotation ‐11.2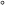 and displacement 15.3 mm, and the rightmost gait $\left(0,\theta^{*}\right)$ averages rotation 15.0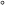 and displacement 5.9 mm. The performances of these gaits qualitatively match or exceed those of the original version [16], though quantitative measurements are not directly comparable due to different ground surface materials.

**TABLE 1:** The 2D displacement (mm) and rotation (degrees) per gait cycle.

$\theta_{L}$		$\theta_{R}=0^{\circ }$	$\theta_{R}=\theta^{*}/2$	$\theta_{R}=\theta^{*}$
$0^{\circ }$	δ [mm]	no	4.3 (1.0)	5.9 (1.6)
	$\gamma \;{[}^{\circ }]$	motion	4.0 (19.8)	15.0 (48.4)
$-\frac{\theta^{*}}{2}$	δ [mm]	12.8 (1.5)	20.0 (1.7)	22.0 (1.6)
	$\gamma \;{[}^{\circ }]$	−9.4 (10.9)	−2.3 (10.5)	6.1 (8.3)
$-\theta^{*}$	δ [mm]	15.3 (2.1)	20.5 (2.1)	21.7 (1.7)
	$\gamma \;{[}^{\circ }]$	−11.2 (8.8)	−4.9 (12.0)	0.1 (7.3)

Values are reported in the notation “mean (standard deviation)”.

**FIGURE 9:**
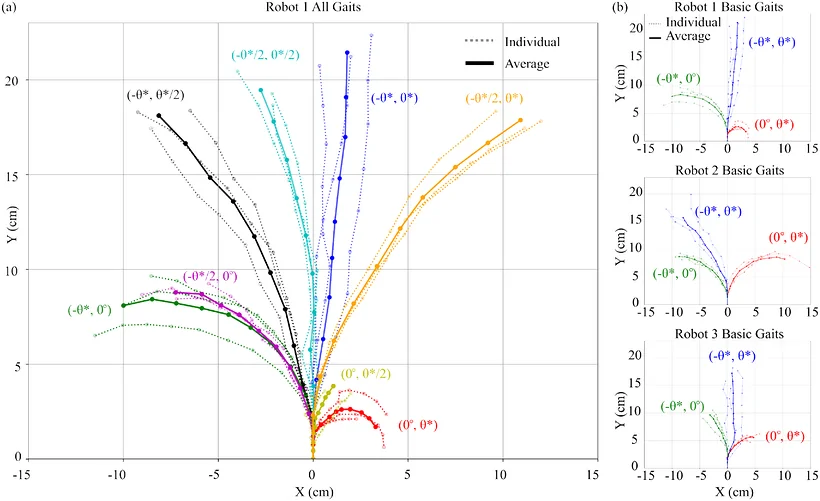
Gait trajectories, based on the position of the center of mass at the end of each gait cycle, 11 cycles per trial. The dotted trajectories are individual trials, while the solid trajectories are the average over the three trials. (a) Trajectories from one robot with the 8 gaits from $\theta_{L}\in \left\{-\theta^{*},-\theta^{*}/2,0^{\circ }\right\}$ and $\theta_{R}\in \left\{0^{\circ },\theta^{*}/2,\theta^{*}\right\}$. (b) Trajectories from three different robots with the three extreme gaits.

To characterize motion within a gait cycle, we plot the center of mass trajectory within a forward gait $\left(-\theta^{*},\theta^{*}\right)$ (broken down into vertical, forward, and sideways motion) and the forward displacement of the back center of the robot. We also identify portions of the cycle when the center of mass is falling or rising, which occurs during state transitions.^4^ Figure 10 shows this motion as the mean and standard deviation across the cycles of a representative run (robot 1 forward gait trial 1).

**FIGURE 10:**
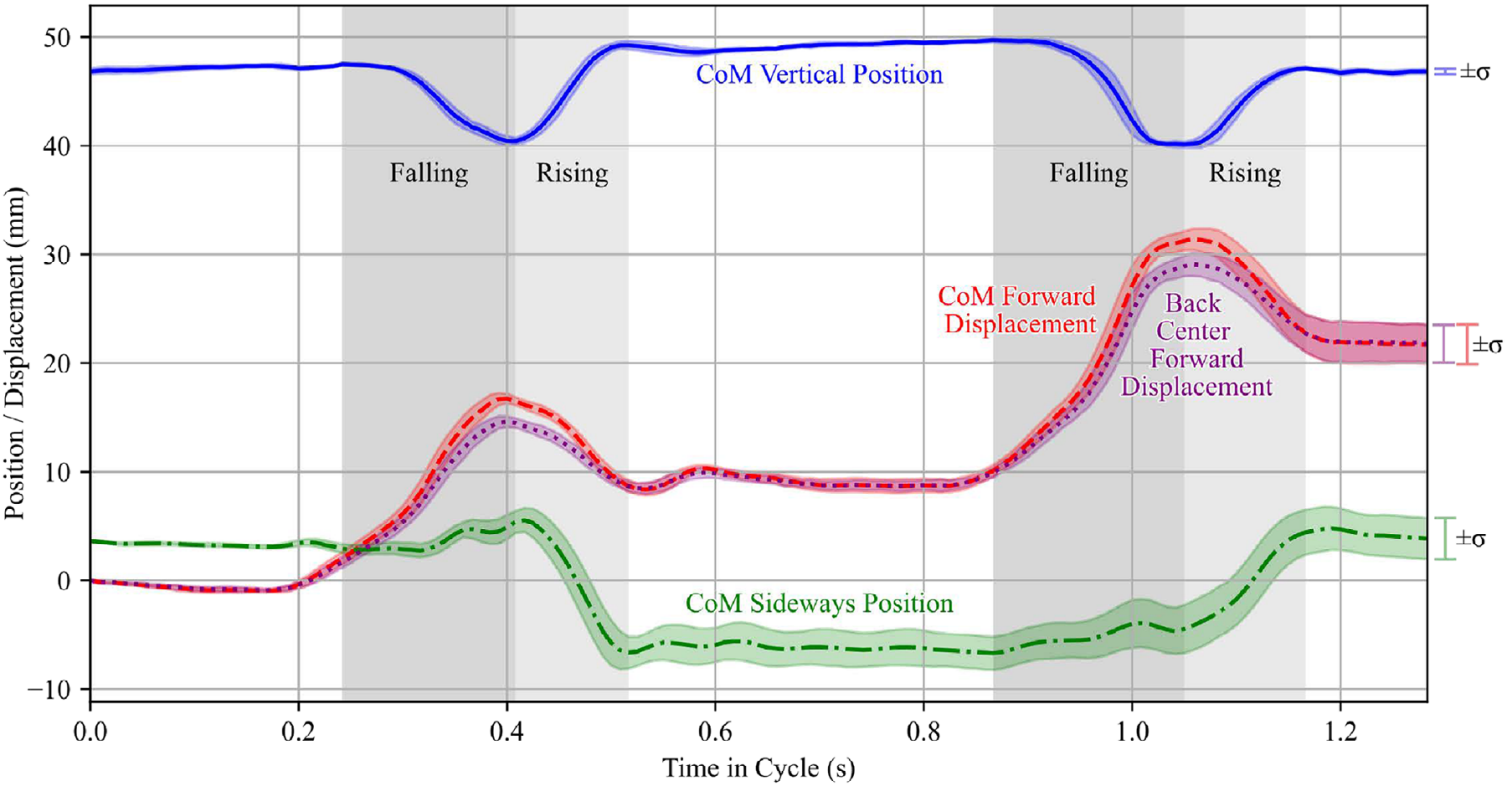
Mean trajectory of the center of mass (CoM) across cycles within a run, broken down into vertical, forward, and sideways components, along with the forward displacement of the back center. Forward is defined for each cycle based on the previous cycle's endpoints. The shadows around each trajectory depict the standard deviation σ, while the background shading identifies when the center of mass is falling or rising during state transitions. The vertical trajectory is the position relative to the ground plane. The forward trajectories are relative to the cycle start, showing accumulated displacement through the cycle. The sideways trajectory is relative to the median within the cycle, showing the approximately symmetric oscillation about the forward direction.

The behavior of the robot is consistent with standard slip‐stick motion common in small‐scale robots (see, for example [80]). Although the motion of the robot is geometrically symmetric in the falling and rising phases, high forward displacement occurs in the slower falling phase compared to the faster rising phase. In the falling phase, the robot flattens out and the back foot maintaining contact with the ground pushes the robot forward. In the rising phase, the robot slips on the ground so that the net backward displacement is smaller. This slippage is due in part to the higher speed of the rising motion and in part to the shifting of the robot's center of mass between the left and right sides.

#### 5.2.3 Performance Consistency

To measure consistency across fabricated instances of the robot, three robots are tested using the extreme left‐turning, forward, and right‐turning gaits: $\left(-\theta^{*},0^{\circ }\right)$, $\left(-\theta^{*},\theta^{*}\right)$, and $\left(0^{\circ },\theta^{*}\right)$. Trajectories are plotted in Figure 9b. Such variation is consistent with the design challenges described above: small differences in the manual assembly steps can affect tendon alignment, mass distribution, and local stiffness, and thus change the robot's bending and ground contact. Note that different types of variation have different sources. Variation between robots, particularly in angular steering bias, is caused by differences introduced during manual assembly. Angular steering bias within an individual robot may similarly arise from fabrication asymmetries. In contrast, the high standard deviation within each gait of each robot is associated with chaos in the slip‐stick ground interaction, along with slight chattering in the servomotor control. However, all three robots are able to crawl and steer, indicating the robustness of the integrated CurveQuad design. Furthermore, as we see in Section 5.2.4, closing the control loop with embedded sensing can overcome gait variance to achieve reliable behaviors.

#### 5.2.4 Embedded Sensing Demonstrations

To demonstrate that the FPCB integration successfully embeds sensing into the body sheet, we conduct several experiments using feedback from the light sensors to steer. The locomotion primitives consist of the forwardmost, leftmost, and rightmost gaits. We implement several behaviors: light‐seeking, path following, following a light gradient, and pursuit‐evasion with multiple robots. Here, we focus on two representative embedded‐sensing demonstrations: light‐seeking by a single robot and pursuit–evasion with multiple robots. Demonstrations of additional feedback‐control behaviors are provided in Section S4: single‐robot path‐following is shown in Figure S3 and Movie S6, while multi‐robot light‐gradient following is shown in Figure S4 and Movie S8.

The sensing demonstrations are conducted on a flat MDF surface with trajectories recorded by vision color‐tracking. Additional tracking details are provided in Section S4.1. In all experiments, raw readings from each light sensor are shifted by a manually calibrated constant to account for baseline differences between sensors.

The light‐seeking experiment is conducted with an LED strip placed vertically at the center of one wall of a 60 cm × 60 cm enclosure blocking off external light. The robot repeatedly selects the extreme left‐turning $\left(-\theta^{*},0^{\circ }\right)$, right‐turning $\left(0^{\circ },\theta^{*}\right)$, or forward $\left(-\theta^{*},\theta^{*}\right)$ gait based on the relative readings of the front‐left and front‐right sensors at the beginning of each cycle. This steers the robot toward the light source over time, working successfully from a variety of starting positions and orientations where the light is detectable from the robot's front. The paths are depicted in Figure 11a, and the full experiment is shown in Movie S5.

**FIGURE 11:**
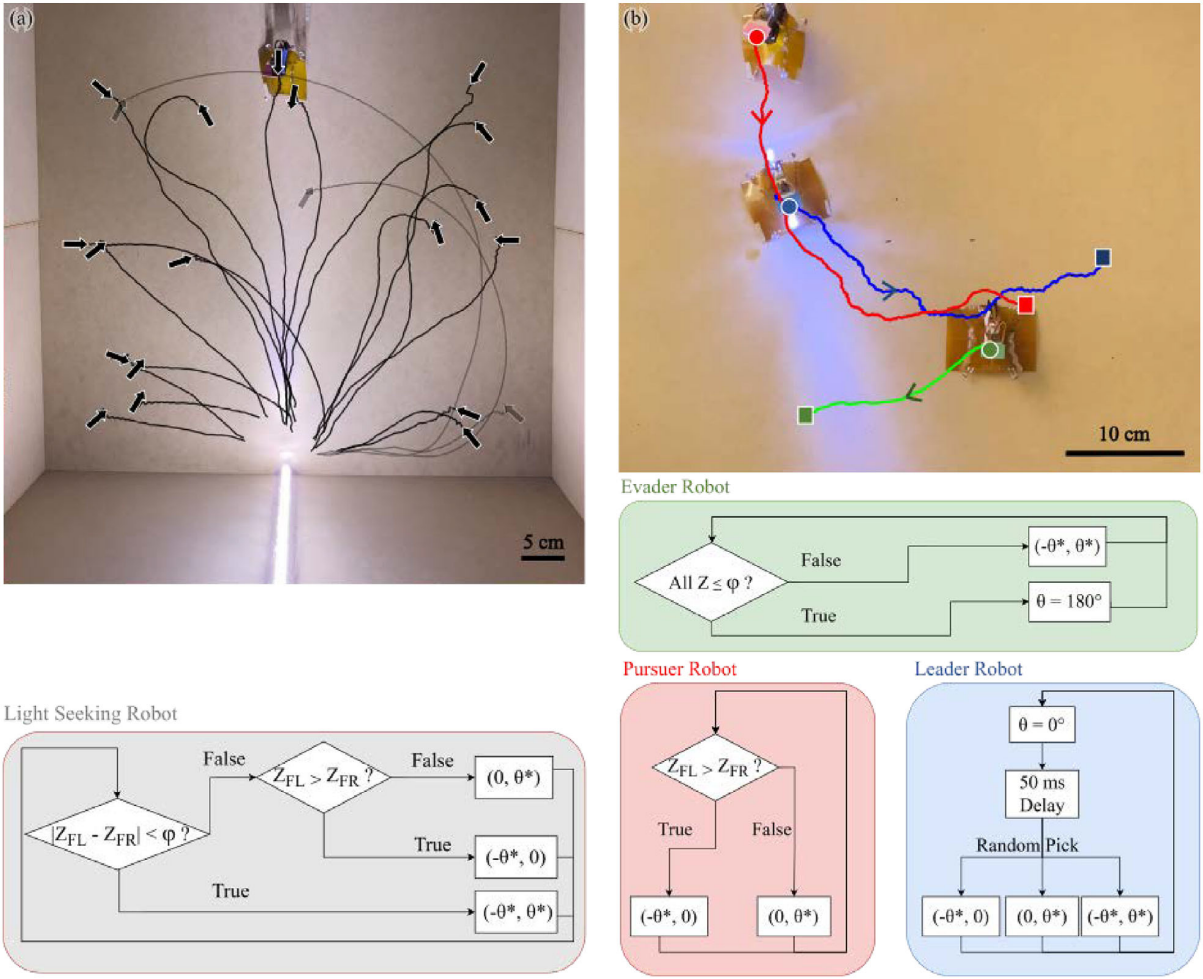
Trajectories and control flowcharts for the embodied sensing experiments. In the flowcharts, $Z_{FL},Z_{FR},Z_{BL},Z_{BR}$ are respectively the front‐left, front‐right, back‐left, and back‐right sensor readings, while φ is a significance threshold. (a) Trajectories of robots originating from various initial positions and orientations (shown by the arrows) and moving toward the light source. The robot repeatedly turns in one direction until it faces the light directly. The semi‐transparent trajectories hit the walls while executing the task, but still reach the light. (b) Trajectories of three robots executing a pursuit‐evasion task. The leader robot (blue, middle) moves randomly, the pursuer (red, top) follows the leader, and the evader (green, bottom) moves away from detected light. Round markers indicate starting locations and rectangular markers indicate ending locations.

In several trials, the robot contacts the enclosure walls during light seeking, which temporarily impacts its trajectory but does not prevent it from continuing the gait and eventually reaching the light source. The robot is already oriented generally toward the light when its side contacts the wall containing the light source, so the contact merely aligns the robot toward the target. The contact force is not sufficient to damage the robot or cause it to tip over. Outside of these light‐seeking tests, we have found that if CurveQuad approaches a wall at a more head‐on orientation, it may get stuck pushing against the wall and can occasionally tip over backward. Since the robot is light, tipping over does not damage it, and it is able to continue walking after being re‐righted.

To increase the complexity of the sensing task, we program three robots to execute a pursuit‐evasion interaction. Each robot is assigned one of three roles: leader, pursuer, or evader. The leader is equipped with two additional LEDs powered directly from the battery and operating independently of the control circuit. At each gait cycle, the leader selects one of the basic gaits at random: $\left(\theta_{L},\theta_{R}\right)=\left(-\theta^{*},0^{\circ }\right)$, $\left(0^{\circ },\theta^{*}\right)$, or $\left(-\theta^{*},\theta^{*}\right)$. The pursuer is programmed to orient toward the light source and follow the leader, while the evader moves away from detected light. To ensure that the pursuer and evader can respond in time, the leader moves more slowly by breaking each commanded servo motion into 20 incremental steps with a 10 ms delay between steps. The trajectories are depicted in Figure 11b, and the full experiment is shown in Movie S7. The evader remains still until the leader approaches, then moves away from the other two robots, while the leader and pursuer follow similar trajectories.

These experiments show successful embedding of light‐sensing in the FPCB CurveQuad body and implementation of feedback control, enabling effective steering despite moderate gait variations within and between robots. The multi‐robot experiments further demonstrate that CurveQuad can respond in real time to changing sensor inputs while executing either shared (light gradient following) or distinct (pursuit‐evasion) behavior rules in a shared workspace. This highlights the potential for collective behaviors and swarm applications leveraging the mass‐manufacturability of FPCBs.

## 6 Transferable Design Principles for FPCB Mechanisms and Origami Robots

Our results highlight key interdependencies in using FPCBs to integrate electronics into compliant mechanisms. Specifically, these principles are likely to be relevant in mechanisms whose functionality relies on sensitive or complex bending of a body sheet, such as robots leveraging non‐rigid‐foldable origami patterns.

### 6.1 Explore Circuit Effects on Stiffness With Increasing Fidelity and Complexity

The first lesson is that iterating over designs with increasing fidelity and complexity enables progressively refining an understanding of the effects of a circuit on mechanical functionality, and thus adds structural complexity to the FPCB only as needed.

In general, an FPCB inherently involves non‐uniform stiffness distributions in the form of traces, stiffener, and surface mount components, all of which critically affect the deformation of an origami pattern and, as a result, the final robot functionality. The stiffness distribution is affected by the overall layer stackup (number and thickness of layers), the trace layout (paths and widths), and the component placement (sizes, mounting types, and locations).

In our design process, we began with a design with separated electronics and uniform body stiffness (Figure 5a, our previous work [16]) and conducted a series of experiments using circuit proxies to approximate the stiffness (Section 3.2.2), then added structural features to the FPCB as needed to escape identified problems: copper pads at the feet to stabilize ground contact, a second copper layer to concentrate trace stiffness in an acceptable region, and double‐sided component mounting and then an extension tail to further concentrate rigid components (Section 3.2.3).

Stepping back to other FPCB mechanisms in which functionality is sensitive to stiffness distribution, this suggests a process beginning with a relatively simple FPCB and low‐fidelity stiffness proxies for exploration, and then iteratively increasing the FPCB complexity and fidelity while refining understanding of the relationship from circuit design to stiffness to functionality. This strategy introduces new structural complexity to the FPCB (e.g., new layers or board extensions) only as needed to escape performance‐critical constraints as they are identified. In the future, integrating simulations such as FEM models for predictive analysis and design would enhance and streamline these placement steps, allowing consideration of more circuit designs and potentially automatic optimization.

### 6.2 Distribute Stiffness Based on Curvature

The second lesson is that in interpreting results from one iteration to decide what to try next, a key to understand stiffness relationships is to compare local curvature between versions.

In our design process, while exploring the relationship between stiffness and performance, the key observation used for design changes was changes in the sheet's curvature throughout its motions. For CurveQuad's functionalities, comparing curvature clarified that stiffness is beneficial when it supports force transmission or ground contact, but harmful when it constrains the high‐curvature regions needed for folding and twisting. As early FPCB‐body versions failed to move their body and feet sufficiently for locomotion (Figure 5b,g), we identified the stiffness of the central panel as the problem by comparing the bending curvature to the versions without the circuit embedded (Figure 5a,d) and over various stiffness proxies (Figure 5e,f,h). In the end, the most successful design strategy was to keep high‐curvature regions compliant while allowing or adding stiffness in regions where deformation was less critical.

Our design process explored relationships between curvature and several other factors, but we expect other FPCB origami robots to encounter additional interactions depending on the order of design exploration. At a high level, the curvature is driven by the crease pattern and the actuation system: we considered those fixed and adjusted the FPCB accordingly, but in other contexts it may be fruitful to adjust those designs alongside the FPCB layout to achieve shape or stiffness goals.

### 6.3 Common Failure Modes Center Around Rigid‐Soft Boundaries

The third lesson is that rigid‐soft boundaries in FPCBs require particular attention to ensure robust performance. Soldered connections of rigid components to flexible bodies are prone to cracking in ways that can be time‐consuming to detect and debug. This is particularly true for large rigid components when placed in areas with higher bending, and for components frequently touched during operation and maintenance.

Throughout our iterations, we observed a number of fatigue‐related failures and maintainability issues in portions of the design experiencing sharp boundaries in compliance. The most frequent failures were at the switch and the JST receptacle headers for the servo and battery wires. Mounting them on top of the servomotor, where they are braced against its rigid case, and adding in an extension tail for added slack dramatically reduced breakage. Meanwhile, we found that smaller electronic component packages were less likely to lose electrical continuity under repeated deformation, likely because they introduced smaller rigid area and shorter solder joints on the flexible substrate. After we switched several components (most importantly the microcontroller) to surface‐mount packaging without leads, we observed fewer disconnections. We also avoided placing rigid components directly on regions that undergo large bending or folding whenever possible. When such placement could not be avoided, we oriented the component and solder joints so that they deform with the folding direction rather than resisting it, helping maintain electrical continuity over repeated folding cycles.

Other origami FPCB mechanisms include features with additional types of abrupt compliance boundaries that do not appear in our designs, such as traces across creases or boundaries between compliant features and rigid regions. We expect designing those to also need attention to fatigue issues.

### 6.4 Electrical Layout is a Combined Electrical and Mechanical Design Problem

Taken together, these design anecdotes demonstrate that when integrating electronics into compliant mechanisms, electrical and mechanical subsystems cannot always be modularized with their interactions captured by a few parameters, as is often assumed in co‐design models [81, 82, 83, 84]. Rather, direct interactions between circuit layout, body compliance and reliability, and design functionality mean that the electrical design inherently involves a mechanical design problem at a lower level than the more typically‐considered higher‐level electromechanical interactions (e.g., the performance, weights, and power draw/supply of sensors, actuators, computers, and batteries). Thus, proper design of fully integrated origami robots must consider the combined mechanical effect of the electrical, sensorimotor, and crease‐pattern design of the robot in order to achieve robust and efficient functionality.

## 7 Discussion and Conclusions

This paper discusses how a flexible printed circuit board can serve as a mechanically active robot body with complex bending behaviors. Through the FPCB‐integrated CurveQuad, we show that the same manufactured sheet can define the curved‐crease structure, route electrical signals, carry sensors and control electronics, provide local stiffness features, and support the deformation required for deployment and locomotion.

The resulting robot deploys from a planar sheet, crawls and steers with a single servomotor, and uses embedded photoresistors for light‐guided behavior. These experiments validate that integrating electronics into the body sheet, when designed with attention to the coupled electrical‐mechanical constraints it introduces, does not prevent the curved‐crease mechanism from folding, twisting, or producing controllable ground interaction. The gait and surface tests further show that locomotion emerges from the coupled effects of body deformation, mass distribution, local stiffness, and contact conditions.

The current limitations of CurveQuad point to future challenges in electronics integration and electrical‐mechanical interactions. First, enabling more complex collective behaviors will likely require additional sensing and communication capabilities, which will need to be embedded while respecting mechanical functionality. Second, the fabrication asymmetries between robots arise from steps within our lab (laser‐cutting the crease arcs with hand‐aligned sheet placement, and manually attaching the servo, electrical components, and link‐tendon system) that could instead be automated in production to improve performance consistency and support mass manufacturing: the design has components vertically attached in order to enable pick‐and‐place assembly. Third, the servo takes up a large portion and mass of the robot with a rigid component: replacing this with embedded smart‐material actuators would make the robot much lighter and more flexible, potentially enabling it to navigate varied terrains and obstacles and conduct cooperative tasks involving climbing over one another. Such a redesign would involve new characterization of actuator response, power consumption, durability, locomotion performance, and complex interactions between those considerations.

More broadly, this work highlights the need to treat FPCB layout as part of the mechanical design of origami robots. Trace routing, component placement, connector location, stiffener placement, and mass distribution all influence how the body deforms and moves. Future work developing computational tools for electromechanical codesign of FPCB mechanisms should therefore model these factors and the interactions we have identified.

Despite these interdependencies, our results suggest that FPCBs with complex bending requirements, including non‐rigid‐foldable origami mechanisms, can be successfully designed by strategic iteration centered on progressively refining an understanding of the causes and effects of the board's stiffness distribution. By co‐designing these electrical and mechanical features, FPCBs can provide a practical route toward compact, manufacturable, and electromechanically integrated origami robotic systems.

## Funding

This work was supported in part by the National Science Foundation (NSF) under Grants 1845339 and 2322898, the Johnson & Johnson WiSTEM2D Scholars Program, the Army Research Office (ARO) under MURI Award W911NF1810327, and the Air Force Office of Scientific Research (AFOSR) under Award FA9550‐23‐1‐0337.

## Conflicts of Interest

The authors declare no conflicts of interest.

## Supporting information


**Supporting File 1**: advs78428‐sup‐0001‐SuppMat.pdf.

**Supporting File 2**: advs78428‐sup‐0002‐MovieS1.mov

**Supporting File 3**: advs78428‐sup‐0003‐MovieS2.mov

**Supporting File 4**: advs78428‐sup‐0004‐MovieS3.mov

**Supporting File 5**: advs78428‐sup‐0005‐MovieS4.mov

**Supporting File 6**: advs78428‐sup‐0006‐MovieS5.mov

**Supporting File 7**: advs78428‐sup‐0007‐MovieS6.mov

**Supporting File 8**: advs78428‐sup‐0008‐MovieS7.mov

**Supporting File 9**: advs78428‐sup‐0009‐MovieS8.mov

## Data Availability

The data supporting the findings of this study are available within the article and its Supplementary Information. Additional data are available from the corresponding author upon reasonable request.

## Endnotes

1 Folding in curved‐crease origami inherently induces bending in the surrounding panels [34].

3 This is calculated based on the principal curvature function from [79], evaluated piecewise across the five surface sections separated by the four crease curves.

4 We identify the falling and rising times from the vertical trajectory of the center of mass: we detect the two globally‐lowest local minima, then find the nearest local maxima on each side of these.
