# Supplementary material for: Co‐Design Insights for Mechanically Active Flexible Printed Circuit Boards Through the CurveQuad Origami Robot
Source: Adv Sci (Weinh). 2026 Oct 29:e78428. Online ahead of print. doi: 10.1002/advs.78428 (PMC13720271; doi:10.1002/advs.78428)
Supplement: Supplementary file 1 [file ADVS-9999-e78428-s005.pdf]

Supporting Information: Co-Design Insights for Mechanically Active Flexible Printed Circuit Boards Through the CurveQuad Origami Robot

Xuelin Wu[†] *Daniel Feshbach*[†] *Satviki Vasireddy* *Louis Beardell* *Bao To* *Yuliy Baryshnikov* *Cynthia Sung*[*]

Xuelin Wu, Daniel Feshbach, Prof. Cynthia Sung
General Robotics, Automation, Sensing & Perception (GRASP) Lab, University of Pennsylvania
Philadelphia, PA 19104, USA
Emails: Xuelin Wu: wuxuelin3@gmail.com; Daniel Feshbach: feshbach@engineering.upenn.edu; Cynthia Sung: crsung@engineering.upenn.edu
Satviki Vasireddy
Princeton Day School
Princeton, NJ 08540, USA
Email: satviki2005@gmail.com
Louis Beardell
Episcopal Academy
Newtown Square, PA 19073, USA
Email: louiebeardell@comcast.net
Bao To
The Peddie School
Hightstown, NJ 08520, USA
Email: tothienbao6a0@gmail.com
Prof. Yuliy Baryshnikov
Department of Mathematics and Electrical and Computer Engineering, University of Illinois Urbana-Champaign
Urbana, IL 61801, USA
Email: ymb@illinois.edu
[†]These authors contributed equally to this work.
[*]Corresponding author: Prof. Cynthia Sung, crsung@engineering.upenn.edu.

Overview. This Supporting Information provides geometric definitions, fabrication and assembly details, supplementary experimental procedures, additional validation results, and movie descriptions for the FPCB-integrated CurveQuad robot.

Contents

- Supplementary Note Section S1: Geometry and kinematic definitions
- Supplementary Note Section S2: Fabrication and assembly details
- Supplementary Note Section S3: Supplementary gait experiment details
- Supplementary Note Section S4: Supplementary embedded-sensing experiment details
- Supplementary Note Section S5: MERLIN Simulation Analysis Details
- Supplementary Note Section S6: List of supplementary movies

S1 Geometry and Kinematic Definitions

S1.1 Crease Pattern

The CurveQuad body sheet has width w and length g. The crease pattern follows the geometry used in [16]. The *foot width* f is the distance between the sheet's corners and the ends of the outside creases. The *between-crease width* b is the horizontal distance between the inner and outer creases on each side. The *inner width from center* i is the distance from the center of the sheet to the inner creases. These parameters define the change in x from each parabola vertex to its endpoints, or the *in-crease width*, as $c_w = \frac{w}{2} - f - b - i$. Then the inner-right (IR) and outer-right (OR) creases can be expressed as

$$x = C_{IR}(y) = c_w \left(2y/g\right)^2 + i, \tag{S1a}$$
$$x = C_{OR}(y) = C_{IR}(y) + b, \tag{S1b}$$

and the left inner and outer creases are $C_{IL}(y) = -C_{IR}(y)$ and $C_{OL}(y) = -C_{OR}(y)$, respectively.

The inner creases C_{IL} and C_{IR} are mountain folds, and the outer creases C_{OL} and C_{OR} are valley folds. The parameter values used in the robot are labeled in Figure S1.

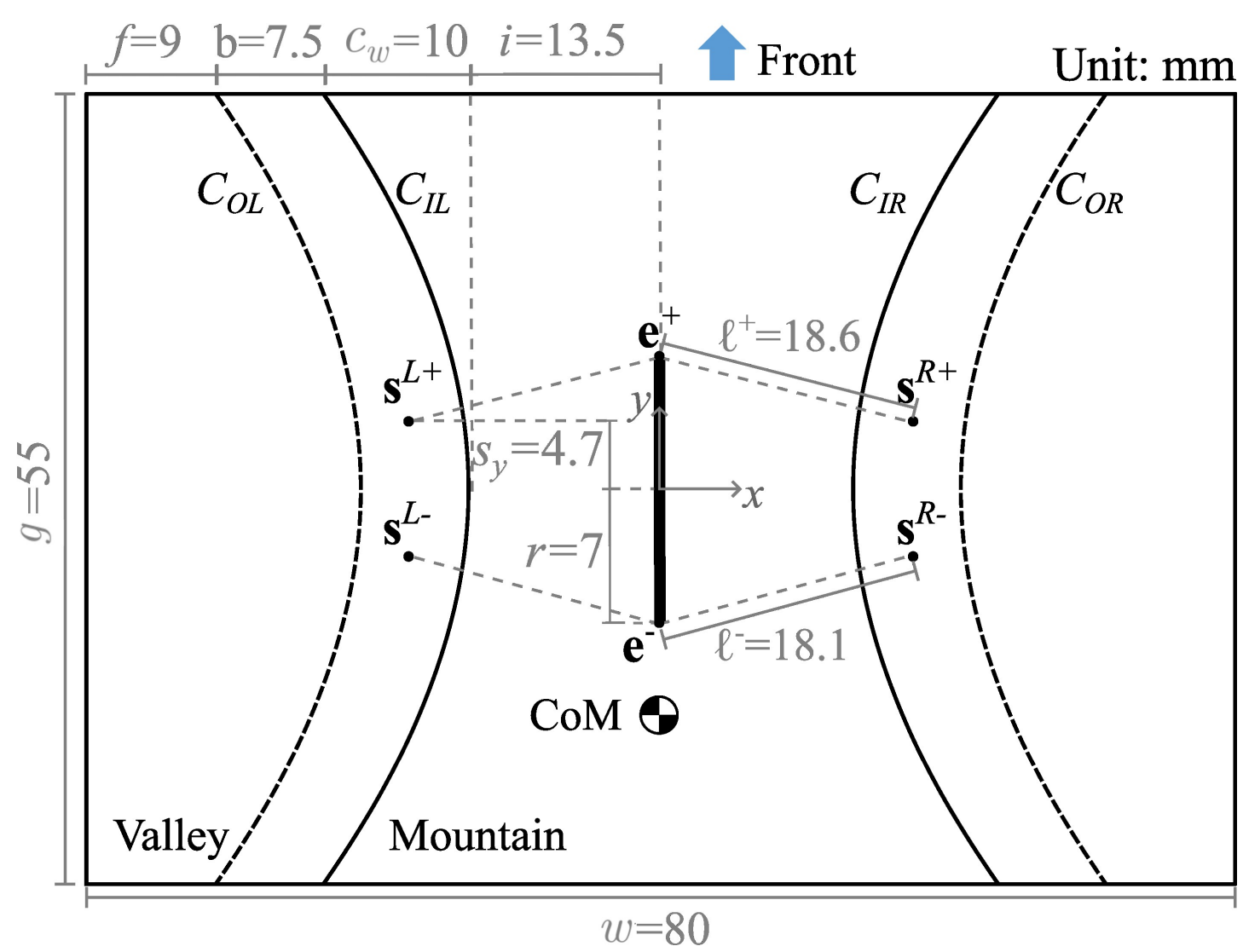

Figure S1. Crease pattern and link-tendon geometry in the unfolded state $\theta = 180°$. The inner creases C_{IL}, C_{IR} are mountain folds and the outer creases C_{OL}, C_{OR} are valley folds. The link is parallel to and below the plane of the sheet, making ℓ^+ and ℓ^- three-dimensional measurements. The sheet plane lies below the center of mass (CoM). The blue arrow indicates the front direction of CurveQuad.

S1.2 Link and Tendon Geometry

The link has effective length $2r$ and rotates about its center, so r is its effective rotational radius.[4] Let the angle of link rotation, measured clockwise from the top view, be denoted by θ. With the link centered on the CurveQuad body, the *link endpoints* $\mathbf{e}^+$ and $\mathbf{e}^-$ are:

$$\mathbf{e}^+(\theta) = (r \sin\theta, -r\cos\theta, e_z^+), \tag{S2a}$$
$$\mathbf{e}^-(\theta) = (-r \sin\theta, r\cos\theta, e_z^-). \tag{S2b}$$

where $e_z^+ < 0$ and $e_z^- < 0$ are the vertical displacements of the tendon attachment points on the link below the robot body sheet. The link orientation in the unfolded state $\theta = 180°$ is shown in Figure S1.

[4] *Effective* length and radius refer to the dimensions affecting the system's kinematics: these are the distances within the plane of rotation between points of connection to external components (tendons and the servomotor). The physical parts implementing the link extend slightly beyond these connection locations.

Each link endpoint is connected to two *sheet attachment points* on the body sheet by tendons: $\mathbf{e}^+$ connects to $\mathbf{s}^{L+}$ and $\mathbf{s}^{R+}$, while $\mathbf{e}^-$ connects to $\mathbf{s}^{L-}$ and $\mathbf{s}^{R-}$. In the unfolded state $\theta = 180°$, the attachment points are

$$\mathbf{s}^{L+}(180°) = (C_{IL}(s_y) - b/2, \quad s_y, \ 0), \tag{S3a}$$
$$\mathbf{s}^{L-}(180°) = (C_{IL}(s_y) - b/2, -s_y, \ 0), \tag{S3b}$$
$$\mathbf{s}^{R+}(180°) = (C_{IR}(s_y) + b/2, \quad s_y, \ 0), \tag{S3c}$$
$$\mathbf{s}^{R-}(180°) = (C_{IR}(s_y) + b/2, -s_y, \ 0). \tag{S3d}$$

where s_y is their common y magnitude. The link endpoints and sheet attachment sites in the unfolded state are shown in Figure S1.

The tendon lengths are ℓ^+ and ℓ^- for the front tendons connected to $\mathbf{e}^+$ and the back tendons connected to $\mathbf{e}^-$, respectively. These lengths are measured with the link at $\theta = 180°$, when the sheet lies flat:

$$\ell^+ = ||\mathbf{s}^{L+}(180°) - \mathbf{e}^+(180°)||, \tag{S4a}$$
$$\ell^- = ||\mathbf{s}^{L-}(180°) - \mathbf{e}^-(180°)||. \tag{S4b}$$

At any link angle θ, the sheet attachment points are constrained by the tendon lengths:

$$||\mathbf{s}^{L+}(\theta) - \mathbf{e}^+(\theta)|| \leq \ell^+, \tag{S5}$$

with symmetric constraints for the other tendons.

For the final robot, $e_z^+ = -5.825$ mm, $e_z^- = -3.825$ mm, $r = 7$ mm, and $s_y = 4.7$ mm. The resulting tendon lengths are $\ell^+ \approx 18.6$ mm and $\ell^- \approx 18.1$ mm.

S1.3 Body States and Gait Definition

The robot body states are defined by the rotation angle θ of the servomotor-driven link. CurveQuad has four key states: *unfolded* ($\theta = 180°$), *folded neutral* ($\theta = 0°$), *folded left* ($\theta \in [-\theta^*, 0°)$), and *folded right* ($\theta \in (0°, \theta^*]$). The $\theta = \pm\theta^*$ states are the left and right extreme states.

To deploy and return toward the flat state, CurveQuad moves between the $\theta = 180°$ state and the $\theta = 0°$ state. In the unfolded state, the tendons are just sufficient in length for the sheet to rest approximately flat. When the link rotates away from this state, the tendons pull the sheet inward at the attachment sites. This creates mountain folds at the inner creases and valley folds at the outer creases. At $\theta = 0°$, the robot reaches the folded neutral state, where the servomotor and battery are lifted away from the ground and the sheet corners act as the feet.

To crawl, the robot twists its body into folded left and folded right states. These states are generated by pulling one diagonal of the body while releasing the other. Body twisting is produced when $\theta \notin \{0°, 180°\}$ because the link is no longer left-right symmetric and therefore pulls one diagonally opposite pair of tendons more than the other pair. For $\theta \in (0°, 90°]$, the diagonal from front-right to back-left is contracted while the other diagonal is released. The opposite occurs for $\theta \in [-90°, 0°)$.

The extreme angle $\pm\theta^*$ is selected to maximize the contraction of one diagonal. Specifically, θ^* is chosen so that the link endpoint is as far as possible from the original (unfolded) position of the sheet attachment point on the contracted diagonal:

$$\theta^* = \underset{\theta \in [0°, 90°]}{\arg\max} \, ||\mathbf{e}^+(\theta) - \mathbf{s}^{R+}(180°)||. \tag{S6}$$

By the triangle inequality, this maximum occurs when the link aligns with the contracted diagonal. Therefore,

$$\theta^* = \arctan\left(\left(C_{IR}(s_y) + b/2\right)/s_y\right). \tag{S7}$$

In our design, this evaluates to $\theta^* \approx 75°$.

A gait is defined by a pair (θ_L, θ_R), where $\theta_L \in [-\theta^*, 0°]$ is the left gait angle and $\theta_R \in [0°, \theta^*]$ is the right gait angle. The basic left-turning, forward, and right-turning gaits are $(-\theta^*, 0°)$, $(-\theta^*, \theta^*)$, and $(0°, \theta^*)$, respectively.

S2 Fabrication and Assembly Details

S2.1 FPCB Circuit Components and Layout

The circuit is powered by a 3.7 V, 70 mAh LiPo battery connected through a JST-2 header. The servomotor is a Turnigy TGY-1370A modified for a 270° range of motion and connected through a JST-3 header. The servomotor is powered directly from the battery, while the rest of the circuit is regulated to 3.3 V through an AP2112K voltage regulator.

The robot is controlled by an ATtiny84A-MUR microcontroller. The FPCB includes a programming connector consisting of six exposed copper pads on the PCB surface. These pads are used to program the microcontroller by temporarily clamping an external Arduino ISP programmer to the board. After programming, the clamp is removed and the robot operates from its onboard battery.

The robot takes sensing input from four PDV-P8001 photoresistors, each wired in series with a 680 Ω resistor. The photoresistors are located at the corners of the central panel and face outward or upward (depending on the sensing needed for a particular task) to detect light gradients.

The circuit also includes two 0603-package surface-mount device (SMD) green LEDs: one programmable LED and one power-indicator LED. The power-indicator LED is wired in parallel with a 1 µF capacitor.

Most components are placed underneath the servomotor or on top of it through a ribbon-like extension tail that curls upward from the back of the FPCB to the top of the servo. The programming pads are placed along the front center so that they can be accessed with a clamp. To minimize interference with body twisting, the traces to the programming pads are concentrated as much as possible along the sagittal (YZ) plane.

Additional copper traces along the back feet act as local stiffeners to increase the surface area of ground contact. These traces are purely mechanical: they are electrically inactive and are not connected to the circuit.

S2.2 Crease Cutting, Tendons, and Servo Horn

The FPCB stackup is manufactured by a commercial supplier (PCBWay). After the FPCB is received, crease and hole cutting are performed in our lab using a PLS4.75 CO_2 laser cutter. The creases are manufactured as a series of alternating-arc cuts, which leave narrow bridges diagonally across the crease lines that bend and twist during folding. The cut pattern is slightly altered from [16], with the outermost partial arcs removed and the arc spacing made precisely regular.

The tendons are laser cut from 0.127 mm PET. The other ends of the tendons connect to the body sheet through holes between the creases. These holes have rounded corners to reduce tearing near the tendon attachment sites. Other than the rounding of the tendon holes, the horn-tendon system is unchanged from [16].

To drive the tendons, the servomotor uses a stacked horn constructed in three parts to prevent tangling. The servo horn is printed from VisiJet SL Clear resin on a ProJet 6000 stereolithography printer. Each part consists of a flat arm with 0.5 mm tall cylindrical spacers. The parts friction-fit together using plus-shaped protrusions and holes 3 mm in diameter and 1.5 mm deep.

The tendons are inserted onto the cylindrical spacers between each pair of horn parts, resulting in two sets of tendons at different heights, as defined in Section S1.2.

The shaft of the servomotor passes through the hole in the middle of the body sheet and connects directly to the horn on the underside of the robot.

The centers of the tendon attachment cylinders are used to calculate the theoretical link radius r. We selected $r = 7$ mm as the longest 1 mm increment that can rotate freely without hitting the sheet. This value is lower than the inner width from the center ($i = 13.5$ mm), because the physical horn takes up space beyond the theoretical link radius and the inner panel of the crease pattern bends inward when the pattern folds.

S2.3 Robot Assembly

The servomotor is attached to the sheet using a rigid mount, slightly modified from the structure in [16] to accommodate components mounted underneath it and the extension tail routed upward through it. The battery is held under the sheet in a case connected to the servo mount behind the body sheet. The servo mount and battery case are 3D printed from PLA on a Prusa MINI+ printer.

After FPCB fabrication, the final assembly process includes soldering the electronic components, attaching the servomotor and battery mount, inserting the tendons into the body slots, connecting the tendons to the servo horn, routing the rear FPCB extension, and connecting the battery, switch, and servomotor.

In the current process, each robot requires approximately 53 minutes of in-house fabrication and assembly, including 2 minutes for laser cutting, 25 minutes for 3D printing, 20 minutes for soldering, and 6 minutes for final assembly.

The final robot is approximately $60 \times 65 \times 50$ mm in the folded state and $80 \times 65 \times 40$ mm in the unfolded state, including the attached electronics. The same robot completed two full battery-discharge sessions, each lasting approximately 55 min, with the battery recharged between sessions. Based on the approximately 1.29 s gait period, this corresponds to over 5000 gait cycles in total, with no observable mechanical failure.

S2.4 Mass Breakdown

The total mass of one FPCB-integrated CurveQuad is 10.938 g. The component-level mass breakdown is listed in Table S1.

Table S1. Mass breakdown for a single FPCB-integrated CurveQuad robot.

Component	Mass [g]
Body sheet with electronics	1.887
Servomotor (Turnigy TGY-1370A)	3.916
Link-tendon mechanism	0.473
Battery (3.7 V LiPo, 70 mAh)	2.085
Mounting, screws, and nuts	2.577
**Total**	**10.938**

S2.5 Material Characterization

Tensile characterization. We performed uniaxial tensile tests on the PET sheet used in the original CurveQuad and the PI stackup used as the FPCB base material without copper. Tests were conducted on an MTS Criterion Model 41 machine. The PET thickness was 0.127 mm and the PI stackup thickness was approximately 0.13 mm. The specimens had a 10 mm wide and 20 mm long gauge section and were tested at a displacement rate of 0.1 mm/s. Force and displacement were converted to engineering stress and strain using the specimen cross-sectional area and gauge length, and Young's modulus was calculated from the slope of the approximately linear region of the stress–strain curve. Two trials were performed for each material. The measured Young's moduli were 2.300 and 2.329 GPa for PET and 1.641 and 1.455 GPa for the PI stackup, corresponding to mean values of approximately 2.31 and 1.55 GPa, respectively. In the second PI trial, clamp slipping was observed at larger deformation, but this occurred after the linear region used for the Young's modulus calculation.

Friction characterization. We also measured friction between each sheet material and the MDF surface used for the gait experiments. Material samples were attached beneath an acrylic carrier and pulled horizontally across the MDF surface under the same applied normal load. The carrier was translated at 5 mm/s, with force recorded at 100 Hz. Five trials were performed for each material. The static friction coefficient was calculated from the initial peak force required to initiate sliding, while the kinetic

friction coefficient was calculated from the approximately steady force during sliding. The resulting average coefficients were $\mu_s = 0.37$ and $\mu_k = 0.22$ for PET and $\mu_s = 0.34$ and $\mu_k = 0.23$ for the PI stackup.

Table S2. Measured material properties of the PET sheet and PI stackup without copper. Friction coefficients were measured against MDF.

Material	Young's modulus [GPa]	μ_s	μ_k
PET	2.31	0.37	0.22
PI stackup	1.55	0.34	0.23

S3 Supplementary Gait Experiment Details

S3.1 Motion-Capture Tracking

Gaits are executed by alternating between the commanded angle pair for 600 ms each to ensure that the servomotor reaches the commanded state. The measured gait period is approximately 1.29 s: we believe the extra 0.09 s is from blocking commands between each cycle. Unless otherwise noted, each gait is measured for three trials of 11 gait cycles each on a medium-density fiberboard (MDF) surface.

Gait experiments are conducted by tracking the robot's center of mass and extracting its motion per gait cycle. The robot is operated in an OptiTrack motion capture system with six Flex13 cameras. A piece of cardstock fixed to the top of the servomotor is registered as a rigid body, and the approximate center of mass is tracked based on its position relative to the servo as estimated from the CAD model.

For data processing, cycles are segmented at gait extremes (these provide a more robust basis for cycle segmentation than the neutral state, because the gait pauses at the extremes but not at the neutral state). Within the horizontal plane, the forward direction for a cycle is defined by the direction vector of the previous cycle's endpoints (i.e., from the previous cycle's start position to this cycle's start position). The first cycle, lacking an orientation baseline, is therefore omitted from our measurements and we process the remaining 10 cycles per trial. Displacements and heading change per cycle are also calculated based on cycle endpoints in the horizontal plane. For the back-center trajectory, the position of the center of the rear edge of the body sheet was obtained from the CAD assembly relative to the estimated CoM and transformed using the same tracked rigid-body pose. In the CAD coordinate system, the back-center point was located at approximately $[0, -18.77, -3.96]$ mm relative to the CoM.

S3.2 Starting-Location Variation

To examine whether local surface variation affected the measured gait behavior, we tested the three basic gaits $(-\theta^*, 0°)$, $(-\theta^*, \theta^*)$, and $(0°, \theta^*)$ from different starting locations on the MDF board. For each gait, we used four starting locations and ran three trials from each location. Each trial contained 11 gait cycles. The trajectories were transformed to have the same starting position and orientation for comparison.

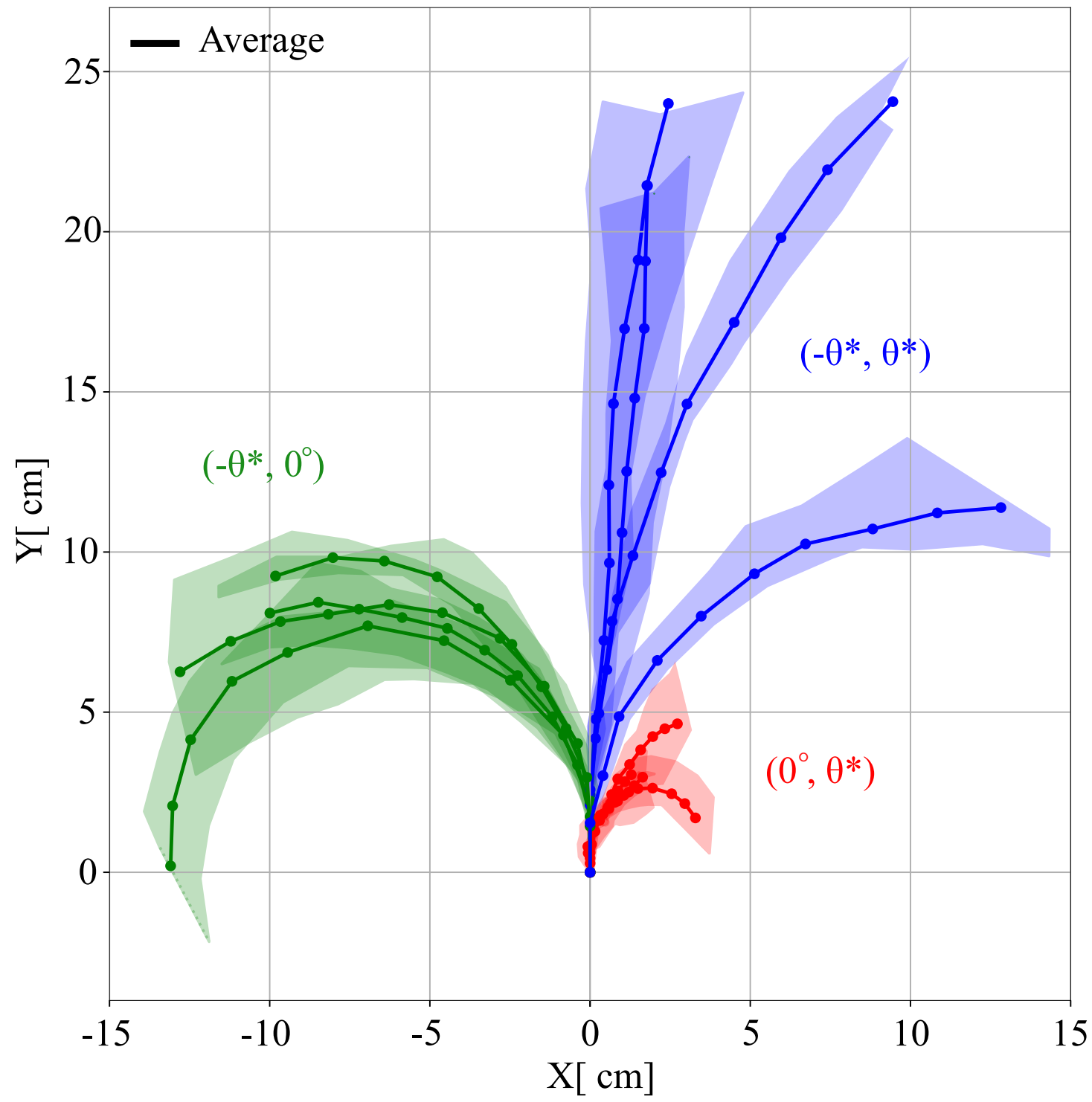

Figure S2. Trajectories produced by one robot executing the three basic gaits from different starting locations on the MDF board. The trajectories are transformed to have the same starting pose. Bold lines indicate the average of three trials, and shaded regions indicate the spread.

S3.3 Surface-Material Variation

We tested the forward gait $(-\theta^*, \theta^*)$ on MDF, paper, metal, acrylic, 120-grit sandpaper, glass, and foam. Each surface was tested in one trial of 11 gait cycles. The displacement and rotation per gait cycle are listed in Table S3.

Table S3. Displacement and rotation of the robot on different materials for the $(-\theta^*, \theta^*)$ gait.

**Material**	δ [mm]	γ [°]
MDF	23.0 (1.7)	0.4 (24.4)
Paper	14.4 (4.5)	4.9 (31.8)
Metal	9.1 (0.5)	2.4 (8.0)
Acrylic	7.7 (1.9)	1.3 (14.2)
120-grit sandpaper	4.8 (1.3)	-1.0 (59.3)
Glass	2.7 (1.2)	1.2 (39.2)
Foam	1.5 (1.4)	-10.6 (144.3)

Values are reported in the notation “mean (standard deviation)”.

S4 Supplementary Embedded-Sensing Experiment Details

S4.1 Video Tracking

For the sensing demonstrations, videos were recorded from an overhead view using an iPhone 13 mini camera at 4K resolution and 30 FPS. Colored paper markers were attached to the top of each robot to facilitate motion tracking. The robot position was computed as the center of the bounding rectangle enclosing the detected region of the corresponding marker color in each video frame.

To smooth the left-right oscillation caused by the robot's gait cycle, the plotted trajectories were filtered using a moving average with a 250-frame window, corresponding to 8.33 s at 30 FPS. In all sensing

experiments, raw readings from each light sensor were shifted by a manually calibrated constant to account for baseline differences between sensors.

S4.2 Path Following

In the path-following experiment, a single robot navigated a projected ring path on an MDF surface. The path had an inner diameter of 25 cm and an outer diameter of 33 cm. It was projected as a white path on a black background to maximize light contrast. The robot used the difference between its two front light sensors to decide whether to steer left or right and remain within the illuminated path.

The same basic left-right light comparison controller was used as in the light-seeking experiment. The robot followed the ring path in both counterclockwise and clockwise directions, as shown in Figure S3.

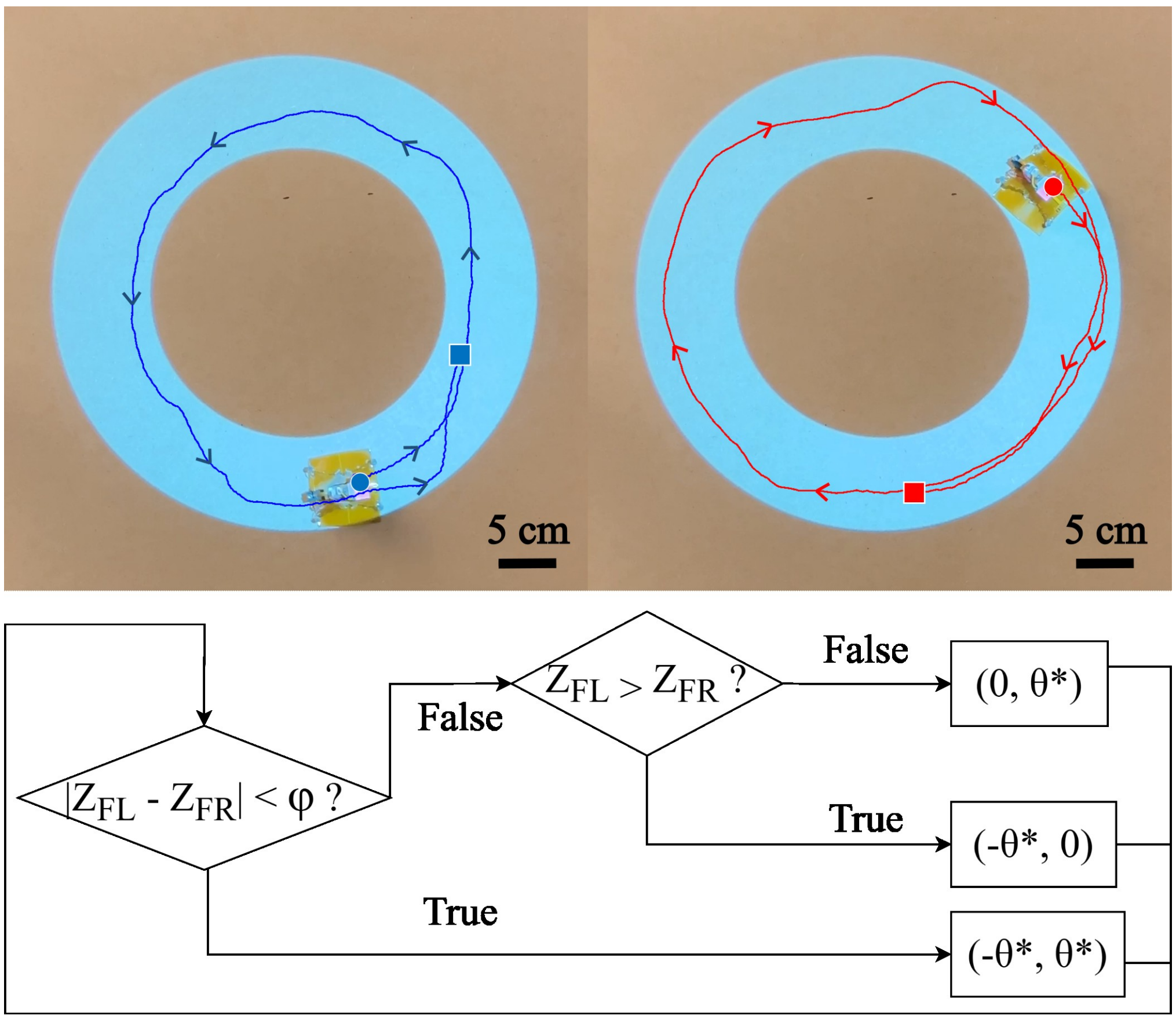

Figure S3. Trajectories of the robot following a projected ring path, moving counterclockwise and clockwise. Round markers show starting locations, and rectangular markers indicate ending locations. The flowchart shows the controller used in this experiment.

S4.3 Following a Light Gradient

In the light-gradient experiment, three robots navigated a projected light-gradient pattern on a 62 cm × 62 cm MDF board. The gradient pattern was designed with discrete brightness levels arranged in rings. The robots were initialized along the horizontal central axis of the board.

The robots were programmed to steer toward increasing brightness and return toward the flat state when they reached a predefined brightness threshold. After the robots returned toward the flat state at the bright regions, the projected gradient pattern was mirrored to change the locations of the brightest regions. The resulting trajectories are shown in Figure S4.

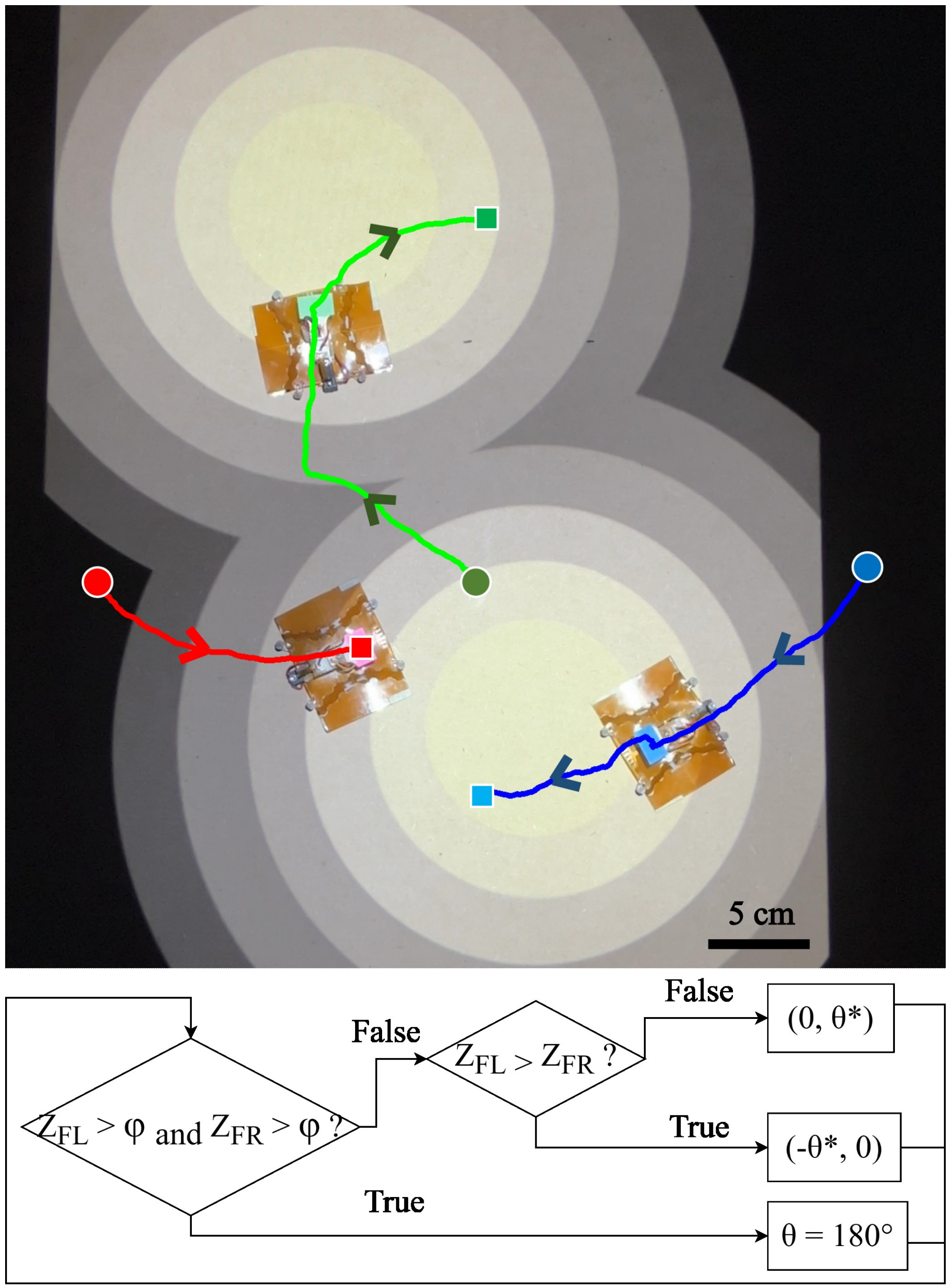

Figure S4. Trajectories of three robots following a projected light gradient. The robots steer toward brighter regions and return toward the flat state once they reach a predefined brightness threshold. Partway through the experiment, the light pattern is mirrored to reactivate the robots. Round markers indicate starting locations, and rectangular markers indicate flat-state locations.

S5 MERLIN Simulation Analysis Details

S5.1 Model Geometry and Simulation Conditions

The CurveQuad body was modeled using MERLIN2 [78], a bar-and-hinge model for origami structures. The processed MERLIN model contained 1021 nodes, 2964 bars, and 1944 triangular elements, with 996 fold hinges and 1872 bending hinges, as shown in Figure S5. In order to create this mesh, the curved crease geometry in Figure 2 was overlaid with a 22x22 grid, subdividing the faces of the pattern to produce a higher resolution mesh. This grid was then imported into MERLIN2's PrepareData function to automatically generate N5B8-type triangulated faces required for the simulation.

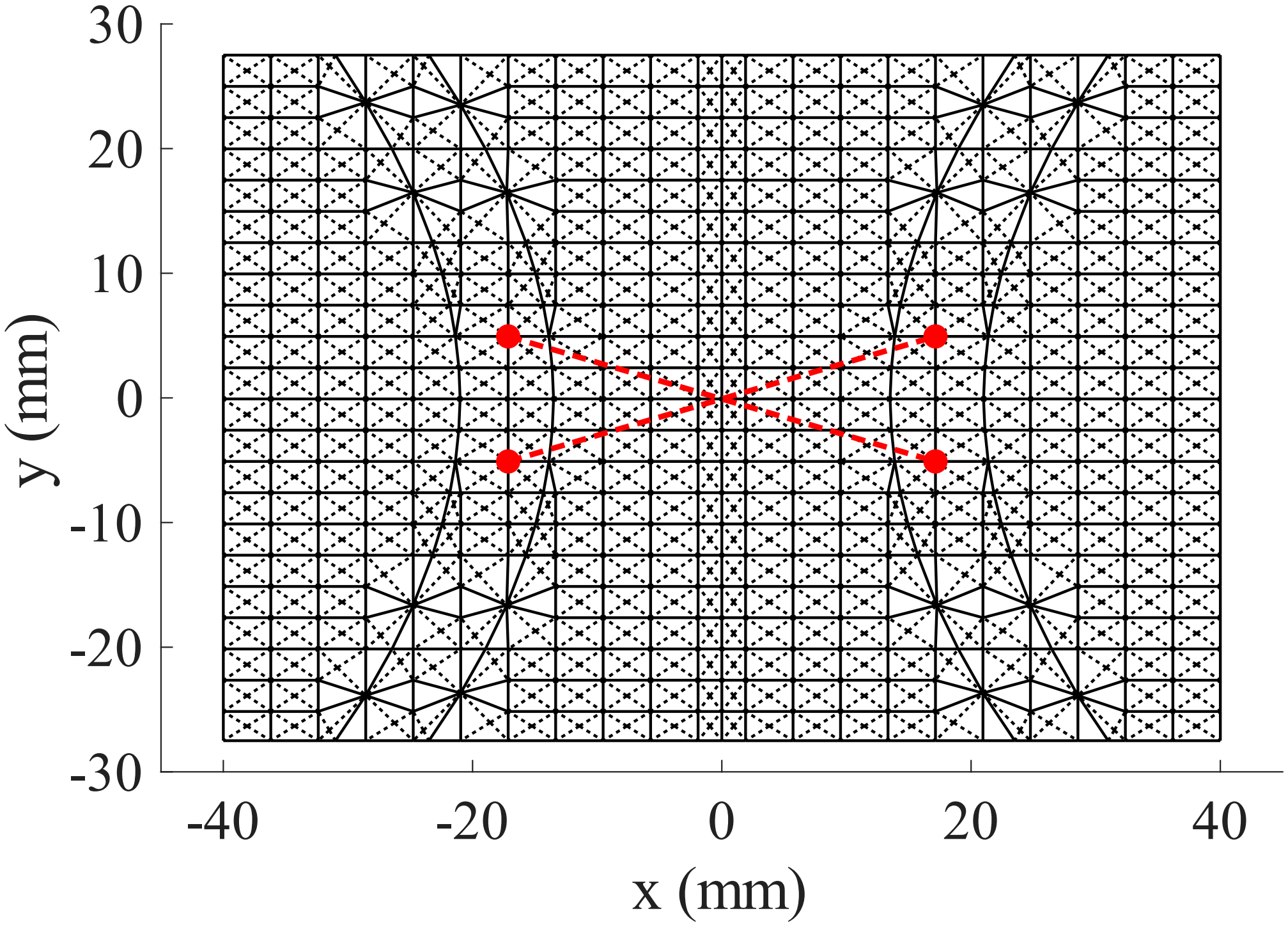

Figure S5. Mesh used for MERLIN2 simulations. Black solid lines indicate original inputted mesh. Black dotted lines indicate bars automatically added by MERLIN2 to triangulate the mesh. Red dots indicate simulated tendon attachment directions, with dashed line indicating the distance used for tendon length calculations.

The baseline material modulus was set to $E_{\mathrm{PI}} = 1.55$ GPa based on the tensile characterization of the PI stackup described in Section S2.5. The Poisson's ratio used in the simulation was 0.35, and the thickness was set to 0.130 mm. Individual hinge elements were modified to have an increased bending stiffness when determined to lie within a copper region, according to the calculation in Section S5.2.

In order to compute the folded state of the robot body, we ran the MERLIN2 simulator in displacement mode. The pattern was fixed at its center node and all of the nodes along the body's centerline were constrained to remain on y axis. The curved folds were assigned to a default fold angle of 30°, corresponding to the slightly folded shape that the robot takes when the tendons are fully released. Then, nodes corresponding to the tendon attachments, depicted in Figure S5, were displaced linearly towards each other to mimic the effect of the tendon actuation system. At each displacement step, the current distance between the nodes was calculated, then compared against the desired tendon length. The required displacement in the tendon direction was computed and then projected onto the x axis. This x displacement was then imposed on the tendon nodes. This approach was chosen in order to maintain the required tendon length constraint while allowing the pattern to freely deform in the y and z directions, as it does on the physical robot. Neutral configurations were simulated by imposing actuation along both tendons, while extreme configurations were simulated by imposing only one.

S5.2 Bending-Stiffness Scaling of Copper-Covered Regions

The MERLIN analysis represents the mechanical effect of the copper layers by scaling the local bending stiffness of the corresponding hinges. The scaling factor is determined from the ratio of the flexural rigidity EI of the Cu/PI/Cu stack to that of the PI-only stack.

For a 1-mm-wide section, the PI-only region has a thickness of 0.130 mm. Its second moment of area about the neutral axis is

$$I_{\mathrm{PI}} = \frac{(1\ \mathrm{mm})(0.130\ \mathrm{mm})^3}{12}. \tag{S8}$$

Where copper is present on both sides, each copper layer is 0.018 mm thick, giving a total stack thickness of 0.166 mm. Because the Cu/PI/Cu stack is symmetric, its neutral axis remains at the mid-plane of the PI layer. The PI layer therefore extends from −0.065 to +0.065 mm from the neutral axis, where

$$0.065\ \mathrm{mm} = \frac{0.130\ \mathrm{mm}}{2}. \tag{S9}$$

Each copper layer extends from 0.065 mm to 0.083 mm from the neutral axis, where

$$0.083\ \mathrm{mm} = 0.065\ \mathrm{mm} + 0.018\ \mathrm{mm} = \frac{0.166\ \mathrm{mm}}{2}. \tag{S10}$$

The combined second moment of area of the upper and lower copper layers is therefore

$$I_{\mathrm{Cu}} = 2(1\ \mathrm{mm})\frac{(0.083\ \mathrm{mm})^3 - (0.065\ \mathrm{mm})^3}{3}. \tag{S11}$$

The flexural rigidity of the PI-only region is

$$(EI)_{\mathrm{PI}} = E_{\mathrm{PI}} I_{\mathrm{PI}}, \tag{S12}$$

while the flexural rigidity of the copper-covered region is

$$(EI)_{\mathrm{Cu/PI/Cu}} = E_{\mathrm{PI}} I_{\mathrm{PI}} + E_{\mathrm{Cu}} I_{\mathrm{Cu}}. \tag{S13}$$

Using $E_{\mathrm{Cu}} = 110$ GPa [85] and the experimentally measured $E_{\mathrm{PI}} = 1.55$ GPa gives

$$(EI)_{\mathrm{PI}} \approx 0.284\ \mathrm{N\,mm^2}, \tag{S14}$$

and

$$(EI)_{\mathrm{Cu/PI/Cu}} \approx 22.08\ \mathrm{N\,mm^2}. \tag{S15}$$

The bending-stiffness scale factor applied to the copper-covered regions in the MERLIN model is therefore

$$S = \frac{(EI)_{\mathrm{Cu/PI/Cu}}}{(EI)_{\mathrm{PI}}} \approx 77.8. \tag{S16}$$

Thus, the bending stiffness of hinges representing copper-covered regions is scaled by approximately 77.8 relative to the PI-only baseline. This is a simplified flexural-rigidity-based representation of the Cu/PI/Cu stack rather than a full laminated-plate mechanics model.

S5.3 Stiffness-Distribution Cases

The simulation cases are summarized in Figure 6 and Table S4. Percent stiffened area is reported as the total volume of the stiffened beams divided by the total volume of the beams in the central panel between the two inner curved creases, ignoring the creases themselves and the outer boundaries. All stiffened cases have a stiffening percentage between 30% and 37%. Priority was placed on ensuring stiffening pattern symmetry over achieving exactly the same percentage across all cases. Variations in the percentages are due to minor differences in the face sizes involved in each case, particularly close to the curved creases. All cases other than the PI-only baseline use the same stiffness multiplier described in Section S5.2.

Table S4. Stiffness distributions evaluated using the MERLIN model.

Case	Stiffening percentage
None	0%
Horizontal stripes	32.1%
Vertical stripes	30.6%
I shape	34.5%
+ shape	31.2%
Grid	36.5%

S6 Supplementary Movies

Table S5. List of Supplementary Movies.

Movie	Description
Movie S1	Self-Folding and Walking from Four Views.
Movie S2	Robot Assembly.
Movie S3	Robot Programming.
Movie S4	Effect of Gait Parameters on Trajectory.
Movie S5	Steering Towards a Light Source.
Movie S6	Path Following.
Movie S7	Pursuit and Evasion.
Movie S8	Following a Light Gradient.

References

[1] D. Rus, M. T. Tolley, *Nature Reviews Materials* **2018**, *3*, 6 101.

[2] S. J. Callens, A. A. Zadpoor, *Materials Today* **2018**, *21*, 3 241.

[3] C. Sung, J. Paik, In *Handbook on Soft Robotics*, 315–344. Springer, **2024**.

[4] D.-Y. Lee, J.-K. Kim, C.-Y. Sohn, J.-M. Heo, K.-J. Cho, *Science Robotics* **2021**, *6*, 53 eabe0201.

[5] W.-H. Chen, S. Misra, Y. Gao, Y.-J. Lee, D. E. Koditschek, S. Yang, C. R. Sung, *IEEE Robotics and Automation Letters* **2020**, *5*, 2 2131.

[6] Z. Zhai, Y. Wang, K. Lin, L. Wu, H. Jiang, *Science Advances* **2020**, *6*, 47 eabe2000.

[7] W.-H. Chen, W. Yang, L. Peach, D. E. Koditschek, C. R. Sung, *IEEE Transactions on Robotics* **2023**, *39*, 2 1260.

[8] A. M. Hoover, E. Steltz, R. S. Fearing, In *IEEE/RSJ International Conference on Intelligent Robots and Systems (IROS)*. **2008** 26–33.

[9] K. Jayaram, J. Shum, S. Castellanos, E. F. Helbling, R. J. Wood, In *IEEE International Conference on Robotics and Automation (ICRA)*. **2020** 10305–10311.

[10] R. M. Bena, X.-T. Nguyen, A. A. Calderón, A. Rigo, N. O. Pérez-Arancibia, *IEEE Robotics and Automation Letters* **2021**, *6*, 4 8173.

[11] S. Felton, M. Tolley, E. Demaine, D. Rus, R. Wood, *Science* **2014**, *345*, 6197 644.

[12] S. Miyashita, S. Guitron, M. Ludersdorfer, C. R. Sung, D. Rus, In *IEEE International Conference on Robotics and Automation (ICRA)*. **2015** 1490–1496.

[13] G. Unger, C. Sung, In *8th International Meeting on Origami in Science, Mathematics, and Education (8OSME)*. **2024** .

[14] D. C. Bershadsky, T. Zhao, G. H. Paulino, E. C. Davidson, *Advanced Functional Materials* **2026**, *36*, 40 e25150.

[15] Z. Zhakypov, K. Mori, K. Hosoda, J. Paik, *Nature* **2019**, *571*, 7765 381.

[16] D. Feshbach, X. Wu, S. Vasireddy, L. Beardell, B. To, Y. Baryshnikov, C. Sung, In *IEEE/RSJ International Conference on Intelligent Robots and Systems (IROS)*. **2023** 2485–2492.

[17] M. E. W. Nisser, S. M. Felton, M. T. Tolley, M. Rubenstein, R. J. Wood, In *IEEE/RSJ International Conference on Intelligent Robots and Systems (IROS)*. **2016** 1254–1261.

[18] N. Bao, Q. Liu, M. F. Reynolds, M. Figueras, E. Smith, W. Wang, M. C. Cao, D. A. Muller, M. Mavrikakis, I. Cohen, P. L. McEuen, N. L. Abbott, *Proceedings of the National Academy of Sciences* **2023**, *120*, 19 e2221740120.

[19] M. Kilian, A. Monszpart, N. J. Mitra, *ACM Transactions on Graphics* **2017**, *36*, 4 1.

[20] J. Carlson, J. Friedman, C. Kim, C. Sung, In *IEEE International Conference on Robotics and Automation (ICRA)*. **2020** 10089–10095.

[21] Y. Sun, J. Wang, C. Sung, In *IEEE International Conference on Robotics and Automation (ICRA)*. **2022** 7189–7195.

[22] Z. Zhakypov, C. H. Belke, J. Paik, In *IEEE/RSJ International Conference on Intelligent Robots and Systems (IROS)*. **2017** 5580–5586.

[23] H. Kabutz, A. Hedrick, W. P. McDonnell, K. Jayaram, In *IEEE/RSJ International Conference on Intelligent Robots and Systems (IROS)*. **2023** 8371–8376.

[24] Z. Yang, D. Chen, D. J. Levine, C. Sung, *IEEE Robotics and Automation Letters* **2021**, *6*, 4 7145.

[25] D. Jeong, K. Lee, *Robotica* **2018**, *36*, 2 261.

[26] N. Mahkam, A. Bakir, O. Özcan, *IEEE Robotics and Automation Letters* **2020**, *5*, 3 3923.

[27] G. Unger, S. Shenoy, T. Li, N. Figueroa, C. Sung, In *IEEE International Conference on Robotics and Automation (ICRA)*. **2025** .

[28] M. Askari, C. Karakadioglu, F. Ayhan, O. Ozcan, In *IEEE International Conference on Robotics and Biomimetics (ROBIO)*. **2017** 19–25.

[29] S. D. De Rivaz, B. Goldberg, N. Doshi, K. Jayaram, J. Zhou, R. J. Wood, *Science Robotics* **2018**, *3*, 25 eaau3038.

[30] A. M. Hoover, S. Burden, X.-Y. Fu, S. S. Sastry, R. S. Fearing, In *3rd IEEE RAS & EMBS International Conference on Biomedical Robotics and Biomechatronics (BioRob)*. **2010** 869–876.

[31] A. F. Güç, M. A. İ. Kalin, C. Karakadioğlu, O. Özcan, In *IEEE International Conference on Robotics and Biomimetics (ROBIO)*. **2017** 26–31.

[32] Huffman, *IEEE Transactions on Computers* **1976**, *100*, 10 1010.

[33] J. P. Duncan, J. Duncan, *Proceedings of the Royal Society of London. A. Mathematical and Physical Sciences* **1982**, *383*, 1784 191.

[34] D. Fuchs, S. Tabachnikov, *The American Mathematical Monthly* **1999**, *106*, 1 27.

[35] S. R. Woodruff, E. T. Filipov, *Meccanica* **2021**, *56*, 6 1613.

[36] J. Flores, L. Stein-Montalvo, S. Adriaenssens, *Extreme Mechanics Letters* **2022**, *57* 101909.

[37] T. Tachi, *Proceedings of Transformables* **2013**, 18–20.

[38] N. Lee, S. Pellegrino, In *Spacecraft Structures Conference*. **2014** 1037.

[39] X. Wang, S. Li, J.-C. Chang, J. Liu, D. Axinte, X. Dong, *Nature Communications* **2024**, *15*, 1 6296.

[40] K. Jayaram, R. J. Full, *Proceedings of the National Academy of Sciences* **2016**, *113*, 8 E950.

[41] X. Ren, Y. Huan, M. Cianchetti, S. Wang, P. Dario, G. Ciuti, *IEEE Transactions on Medical Robotics and Bionics* **2024**.

[42] W. Ma, B. Li, L. Jiang, Y. Wu, R. Bai, G. Chen, *Advanced Intelligent Systems* **2024**, 2300828.

[43] S.-H. Chae, S.-M. Baek, J. Lee, K.-J. Cho, *IEEE/ASME Transactions on Mechatronics* **2022**, *27*, 6 5890.

[44] J.-S. Koh, K.-J. Cho, *IEEE/ASME Transactions on Mechatronics* **2013**, *18*, 2 419.

[45] X. Yang, L. Chang, N. O. Pérez-Arancibia, *Science Robotics* **2020**, *5*, 45 eaba0015.

[46] L. Christensen, N. Fischer, S. Kroffke, J. Lemburg, R. Ahlers, *Journal of Ship Production and Design* **2011**, *27*, 3 127.

[47] T. Ching, J. Z. W. Lee, S. K. H. Win, L. S. T. Win, D. Sufiyan, C. P. X. Lim, N. Nagaraju, Y.-C. Toh, S. Foong, M. Hashimoto, *Science Robotics* **2024**, *9*, 92 eadk4533.

[48] Q. Su, S. Zhang, Y. Liu, J. Deng, *IEEE Transactions on Industrial Electronics* **2020**, *68*, 2 1466.

[49] C. Wang, H. Li, Z. Zhang, P. Yu, L. Yang, J. Du, Y. Niu, J. Jiang, *Journal of Intelligent & Robotic Systems* **2022**, *105*, 3 56.

[50] H. Wei, Y. Zhang, T. Zhang, Y. Guan, K. Xu, X. Ding, Y. Pang, *Space Science Reviews* **2021**, *217* 1.

[51] E. W. Hawkes, L. H. Blumenschein, J. D. Greer, A. M. Okamura, *Science Robotics* **2017**, *2*, 8 eaan3028.

[52] C. Tang, B. Du, S. Jiang, Q. Shao, X. Dong, X.-J. Liu, H. Zhao, *Science Robotics* **2022**, *7*, 66 eabm8597.

[53] V. Iyer, A. Najafi, J. James, S. Fuller, S. Gollakota, *Science Robotics* **2020**, *5*, 44 eabb0839.

[54] M. A. I. Kalın, C. Aygül, A. Türkmen, J. Kwiczak-Yiğitbaşı, B. Baytekin, O. Özcan, *IEEE Robotics and Automation Letters* **2020**, *5*, 3 3854.

[55] D. Zarrouk, R. S. Fearing, In *IEEE International Conference on Robotics and Automation (ICRA).* **2014** 2569–2569.

[56] S. Noji, S. Nansai, N. Kamamichi, H. Itoh, *IEEE Robotics and Automation Letters* **2022**, *7*, 3 6399.

[57] D. Farhadi, L. Pernigoni, D. Melancon, K. Bertoldi, *Advanced Materials* **2025**, *37*, 34 2502293.

[58] J. Ma, J. Peng, Y. Ma, K. Yuan, A. Qin, W. Zhu, X. Chen, *IEEE Robotics and Automation Letters* **2025**, *10*, 8 8011.

[59] X. Huang, K. Kumar, M. K. Jawed, Z. Ye, C. Majidi, *IEEE Robotics and Automation Letters* **2019**, *4*, 3 2415.

[60] S. Kim, R. Kubicek, S. Bergbreiter, *Advanced Functional Materials* **2023**, *33*, 47 2304991.

[61] C. D. Onal, M. T. Tolley, R. J. Wood, D. Rus, *IEEE/ASME Transactions on Mechatronics* **2014**, *20*, 5 2214.

[62] S. Kim, A. M. Johnson, S. Bergbreiter, *Advanced Intelligent Systems* **2024**, 2400196.

[63] Y. Chiu, Y.-Z. Chen, C.-C. Hsieh, H.-C. Hong, *IEEE Sensors Journal* **2021**, *21*, 24 27343.

[64] X. Ji, X. Liu, V. Cacucciolo, M. Imboden, Y. Civet, A. El Haitami, S. Cantin, Y. Perriard, H. Shea, *Science Robotics* **2019**, *4*, 37 eaaz6451.

[65] L. Guo, G. S. Guvanasen, X. Liu, C. Tuthill, T. R. Nichols, S. P. DeWeerth, *IEEE Transactions on Biomedical Circuits and Systems* **2012**, *7*, 1 1.

[66] A. Dementyev, J. Qi, J. Ou, J. Paradiso, In *IEEE/RSJ International Conference on Intelligent Robots and Systems (IROS).* **2018** 6099–6104.

[67] W. Gao, H. Ota, D. Kiriya, K. Takei, A. Javey, *Accounts of Chemical Research* **2019**, *52*, 3 523.

[68] M. Yang, Z. Ye, Y. Ren, M. Farhat, P.-Y. Chen, *Micromachines* **2023**, *15*, 1 26.

[69] J. Lee, Y. Cha, *Smart Materials and Structures* **2017**, *26*, 12 125019.

[70] N. Hou, J. Wen, Y. Pan, M. Taghavi, *Advanced Intelligent Systems* **2024**, 2300738.

[71] C. Sung, D. Rus, *Journal of Mechanisms and Robotics* **2015**, *7*, 2 021012.

[72] Y. Li, W. Liu, Y. Deng, W. Hong, H. Yu, *npj Flexible Electronics* **2021**, *5*, 1 3.

[73] D. Lee, K. Saito, T. Umedachi, T. D. Ta, Y. Kawahara, In *ACM International Joint Conference and International Symposium on Pervasive and Ubiquitous Computing and Wearable Computers (UbiComp).* **2018** 392–395.

[74] A. C. Siegel, S. T. Phillips, M. D. Dickey, N. Lu, Z. Suo, G. M. Whitesides, *Advanced Functional Materials* **2010**, *20*, 1 28.

[75] D.-H. Kim, J.-H. Ahn, W. M. Choi, H.-S. Kim, T.-H. Kim, J. Song, Y. Y. Huang, Z. Liu, C. Lu, J. A. Rogers, *Science* **2008**, *320*, 5875 507.

[76] N. Lu, Z. Suo, J. J. Vlassak, *Acta Materialia* **2010**, *58*, 5 1679.

[77] Z. Zhakypov, J. Paik, *IEEE Transactions on Robotics* **2018**, *34*, 1 151.

[78] K. Liu, G. H. Paulino, et al., In *7th International Meeting on Origami in Science, Mathematics, and Education (7OSME)*. Taylor & Francis Boca Raton, FL, **2018** .

[79] A. Jacobson, et al., gptoolbox: Geometry processing toolbox, **2024**, URL `http://github.com/alecjacobson/gptoolbox`.

[80] C. Pawashe, S. Floyd, M. Sitti, In *Proceedings of Robotics: Science and Systems IV*. Zurich, Switzerland, **2008** .

[81] A. Mehta, J. DelPreto, D. Rus, *Journal of Mechanisms and Robotics* **2015**, *7*, 2 021015.

[82] N. Bezzo, P. Gebhard, I. Lee, M. Piccoli, V. Kumar, M. Yim, In *ASME International Design Engineering Technical Conferences and Computers and Information in Engineering Conference (IDETC/CIE)*, volume 57199. **2015** V009T07A009.

[83] D. Chen, Z. Huang, C. Sung, In *ASME International Design Engineering Technical Conferences and Computers and Information in Engineering Conference (IDETC/CIE)*. **2023** DETC2023–115313.

[84] A. Chattoraj, R. Lin, D. Martinez, A. Mehta, In *IEEE 20th International Conference on Automation Science and Engineering (CASE)*. **2024** 4012–4019.

[85] K. Kammuri, A. Miki, H. Takeuchi, *Journal of Microelectronics and Electronic Packaging* **2017**, *14*, 2 70.
